# Blood-derived microRNA signatures associated with hippocampal structure and atrophy rate: findings from the Rhineland Study

**DOI:** 10.1038/s41380-026-03611-6

**Published:** 2026-04-24

**Authors:** Konstantinos Melas, Valentina Talevi, Mohammed Aslam Imtiaz, Dennis M. Krüger, Tonatiuh Pena-Centeno, André Fischer, N. Ahmad Aziz, Monique M. B. Breteler

**Affiliations:** 1 Population Health Sciences, German Centre for Neurodegenerative Diseases (DZNE), Bonn, Germany; 2 Department for Epigenetics and Systems Medicine in Neurodegenerative Diseases, German Center for Neurodegenerative Diseases (DZNE), Göttingen, Germany; 3 Bioinformatics Unit, German Centre for Neurodegenerative Diseases (DZNE), Göttingen, Germany; 4 Department for Psychiatry and Psychotherapy, University Medical Center Göttingen, Göttingen, Germany; 5 Cluster of Excellence MBExC, University of Göttingen & University Medical Center Göttingen, Göttingen, Germany; 6 Department of Neurology, Faculty of Medicine, University of Bonn, Bonn, Germany; 7 Institute for Medical Biometry, Informatics and Epidemiology (IMBIE), Faculty of Medicine, University of Bonn, Bonn, Germany

**Keywords:** Diagnostic markers, Molecular biology, Genetics

## Abstract

MicroRNAs (miRNAs) have been linked to brain disorders, but their relations with hippocampal structure and atrophy remain unexplored. As the hippocampus is pivotal for cognition and dementia, understanding these relations and their specificity for the hippocampus would elucidate miRNA involvement in brain health and neurodegeneration. Here, using population-based data, we cross-sectionally and longitudinally examined the associations of blood-derived miRNAs with left and right hippocampal volume, hippocampal asymmetry, and total brain volume. Expression of miRNAs and their putative target genes was measured at study baseline in whole blood using RNA sequencing. Brain imaging measures were examined at baseline and re-examined 4.60–8.02 years later using 3 T MRI. We investigated miRNA associations with imaging measures cross-sectionally using linear regression and longitudinally using linear mixed-effect models. Cross-sectionally, six miRNAs (miR-199a-3p, miR-199b-3p, miR-155-5p, miR-146a-5p, miR-6859-5p, miR-505-5p) were associated exclusively with left hippocampal volume. Longitudinally, another five miRNAs (miR-361-3p, miR-4473, miR-381-3p, miR-543, miR-370-3p) were associated with left hippocampal, right hippocampal, and total brain atrophy rates. Twenty-one miRNAs were exclusively associated with total brain atrophy rate. In whole blood, miRNAs identified in the cross-sectional analysis targeted genes related to brain development, memory, and synapse assembly. MiRNAs from the longitudinal analysis targeted genes related to axonal and dendritic growth. Several identified miRNAs were previously linked to neurodegeneration. Especially miR-146a-5p and miR-370-3p have been consistently linked to dementia and could be investigated as presymptomatic blood-based biomarkers. The brain-specific functions and interactions with target genes of identified miRNAs could be further investigated to develop therapeutic strategies against neurodegeneration.

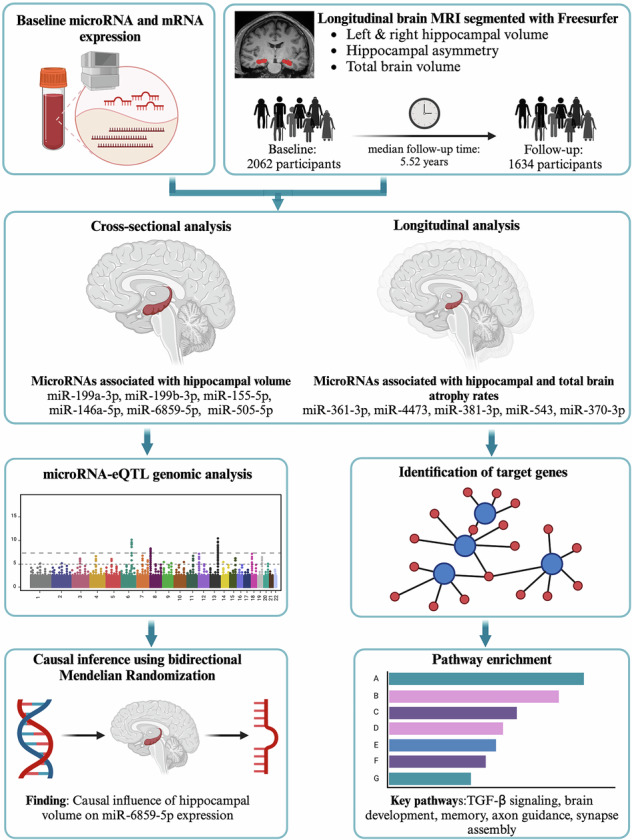

## Introduction

MicroRNAs (miRNAs) are small, non-coding, single-stranded RNAs with a critical role in the epigenetic regulation of neuronal development, function, and pathology [1, 2]. They regulate cellular functions by binding to and inhibiting target genes [3]. As a single miRNA can bind to several target genes, its expression levels can reflect and regulate multiple (patho)physiological processes [4]. Thus, they have been investigated for the diagnosis and therapy of neurodegenerative diseases, especially Alzheimer’s Disease (AD) [4–10]. However, in past studies, neurodegenerative diseases have mostly been dichotomized based on the presence or absence of a diagnosis. Although heuristically useful, this simplification disregards the heterogeneous nature of most such diseases as well as the potential interactions between miRNAs and the brain beyond disease, i.e., both during development and aging [11]. To further elucidate the role of miRNAs in brain health and neurodegeneration, a detailed understanding of their association with key brain-related phenotypes is needed, coupled with a characterization of their biological functions. Moreover, identifying miRNAs associated with phenotypes typical of neurodegeneration in healthy, younger individuals could aid the development of miRNA biomarkers for the very early detection of neurodegenerative diseases.

The hippocampus is essential for long-term memory formation and spatial navigation [12]. Although diffuse brain atrophy is expected during “healthy” aging, hippocampus-specific atrophy due to accumulation of neuropathology is one of the earliest changes observed in AD and other dementias [13–16]. Notably, the left hippocampus tends to be smaller than the right [17] and this asymmetry increases in dementia [18], but there is little evidence for lateralized atrophy in normal aging [19, 20]. Additionally, a higher genetic risk of AD has been associated with a smaller hippocampus [21], which in turn has been associated with future cognitive decline [13, 22] and worse memory performance, even in non-demented, young individuals [23, 24]. Thus, the structure and atrophy patterns of the hippocampus in each hemisphere provide granular information on brain health and neurodegeneration. Previous studies have shown that blood-derived miRNA expression is related to brain atrophy in cohorts consisting of neurologic or psychiatric patients [25–31]. Moreover, we previously showed that some cognition-related miRNAs are cross-sectionally associated with hippocampal volume in the general population [32]. However, our previous study only examined this association in specific, cognition-related miRNAs. The complete relationship of blood-derived miRNAs with hippocampal structure and atrophy in the general population remains unexplored. This relationship is particularly relevant for miRNAs suggested as biomarkers for the early detection of dementia [4], as it would support their involvement in preclinical neuropathology, before the emergence of cognitive symptoms.

Here, we aimed to identify blood-derived miRNAs cross-sectionally and longitudinally related to hippocampal volume and left-to-right asymmetry, considering a decrease in hippocampal volume over time as a proxy of hippocampal atrophy. Our analysis was based on data from the population-based Rhineland Study. To determine whether our findings were specific to the hippocampus, we also identified the miRNAs cross-sectionally and longitudinally related to total brain volume. Subsequently, we employed functional genomics to uncover genes and biological pathways regulated by the identified miRNAs. Moreover, we performed genome-wide association studies (GWAS) to detect miRNA expression quantitative trait loci (miR-eQTLs). Lastly, we leveraged these miR-eQTLs to assess potentially causal associations of miRNAs with imaging measures in a Mendelian randomization framework.

## Methods

### Study design

We based our analysis on data from the baseline and first follow-up examinations of the Rhineland Study, an ongoing prospective, population-based cohort study in Bonn, Germany. All residents of two pre-defined geographical areas of Bonn are invited to participate in the Rhineland Study. Inclusion criteria are age of 30 years or older and sufficient command of the German language to provide informed consent. Both at baseline and follow-up, each participant underwent a comprehensive 7-hour examination protocol.

### Standard protocol approvals, registrations, and participant consent

Approval to undertake the study was obtained from the Ethics Committee of the University of Bonn, Medical Faculty (Lfd. Nr.: 338/15). The study is carried out in accordance with the recommendations of the International Conference on Harmonization Good Clinical Practice standards. We obtained written informed consent from all participants in accordance with the Declaration of Helsinki.

### Analytical samples used for cross-sectional and longitudinal analyses

At study baseline, biomaterial was selected from the first 3000 participants of the Rhineland Study who provided blood samples for miRNA sequencing. Cross-sectional analyses were performed based on a subset of 2062 participants with complete baseline miRNA and hippocampal imaging data. For the longitudinal analyses, we related baseline miRNA data to baseline and follow-up hippocampal imaging data. Complete baseline miRNA and follow-up hippocampal imaging data were available in 1634 participants. The period between baseline and follow-up visits ranged from 4.60 to 8.02 years, with a median of 5.52 years and a total person-time of 12 750 years (Supplementary Figure S1).

### Image acquisition and hippocampal segmentation

Brain imaging was performed both at study baseline and during the follow-up visits, providing measurements of brain volume at two time points. Specifically, eligible participants underwent the same one-hour brain imaging protocol using 3 T MRI scanners (Siemens Prisma Magnetom, Erlangen, Germany) equipped with a 64-channel head-neck coil [33]. T1-weighted images were used to obtain measurements of left and right hemisphere hippocampal volume, total brain volume, and estimated Total Intracranial Volume (eTIV) using the standard Freesurfer processing pipeline (version 6.0) [34, 35]. We evaluated total brain volume to assess whether our findings reflected phenomena across the whole brain or were specific to the hippocampus. To account for differences in head size, hippocampal and total brain volumes were normalized for eTIV using linear regression (see ‘Statistical analysis’). Hippocampal asymmetry was defined as (left hippocampal volume – right hippocampal volume)/(left hippocampal volume + right hippocampal volume).

### Blood sample acquisition and storage

Blood samples were collected after overnight fast between 7:00–9:45 in the morning. For miRNA and messenger RNA (mRNA) sequencing, samples were stored in PAXgene Blood RNA tubes (PreAnalytix/Qiagen) at −80° Celsius. Total RNA was isolated according to manufacturer’s instructions using the PAXgene Blood miRNA Kit, following the automated purification protocol (PreAnalytix/Qiagen) [36].

### MiRNA and gene expression measurement

We performed the miRNA sequencing on the Illumina HiSeq 2000 platform, measured over 44 plates, and the mRNA sequencing on the NovaSeq6000 platform, measured over 28 plates, as detailed before [36]. MiRNA and genes with overall mean expression greater than 15 reads and expressed in at least 5% of the participants were used for further analysis, leading to 415 unique miRNAs and 11 019 unique genes. Raw miRNA and gene counts were normalized and log-transformed before analysis [37]. MiRNA and gene expression were only measured at study baseline.

### Additional variables

We evaluated the overall health and education of participants based on physician-diagnosed diseases (dementia, Parkinson’s disease, multiple sclerosis, hypertension, diabetes, stroke, coronary artery disease), smoking, and educational level. This information was based on self-reports, supplemented by clinical (for hypertension) and laboratory (for diabetes and smoking) measurements. To examine the association of hippocampal volume with cognition, we included a composite global cognition z-score, indicative of participant performance across four different global domains as measured by a neuropsychological battery at study baseline [32]. As potential confounders, in addition to age and sex, we controlled for differential blood cell counts, fasting blood lipid levels, and estimated Glomerular Filtration Rate (eGFR) based on cystatin-C levels. These laboratory variables were measured following standard procedures at the University Hospital Bonn (Supplementary Methods 1).

### Statistical analysis

We compared baseline characteristics between the analyzed subsets of our cohort using type III Analysis of Covariance adjusted for age and sex, except when examining age and sex themselves, where no adjustment was used.

Before further analyses, all numerical variables except age were z-standardized to enable comparison of effect sizes. We used multivariable linear regression to examine the association of left and right hippocampal volume (independent variables) with global cognition (dependent variable), adjusting for age, sex, and eTIV. We also used multivariable linear regression to examine the cross-sectional association of miRNA expression (independent variable) with each imaging measure (dependent variable), adjusting for age, sex, and miRNA sequencing plate (batch). When assessing hippocampal or total brain volume as the dependent variables, we additionally adjusted for eTIV.

For the longitudinal analyses, we examined whether miRNA expression at baseline was associated with changes in imaging measures as a function of time, i.e., the yearly rates of change in hippocampal volume, hippocampal asymmetry, and total brain volume. For hippocampal and total brain volume, we considered these rates as indicative of hippocampal and total brain atrophy. We modeled these associations using linear mixed-effect models as follows:

$${Y}_{i}= {\beta }_{0}+{\beta }_{1}\cdot {age}+{\beta }_{2}\cdot m{icroRNA}+{\beta }_{3}\cdot a{ge}\times {microRNA} +\,{b}_{0,i}+{b}_{1,i}\cdot {age}+{\varepsilon }_{\iota }\,$$

Here, *Y*_*i*_ denotes the imaging measure at any time point for participant *i, β*_*0*_ denotes the fixed mean intercept, *β*_*1*_ denotes the fixed effect of age (i.e., the average change rate of the outcome per year across all individuals), *β*_*2*_ is the fixed effect of baseline miRNA expression, *β*_*3*_ is the fixed effect of the interaction between baseline miRNA expression and age, and *ε*_*i*_ represents the residual error. For each participant, we also included a random intercept (*b*_*0,i*_) to account for inherent between-participant differences of imaging measures at the baseline examinations. Similarly, we included a random slope for age (*b*_*1,i*_) to account for each participant experiencing different levels of yearly change in imaging measures. The effect of baseline miRNA expression on the average change rate of imaging measures was thus given by the interaction term *β*_*3*_. The average change rate associated with one standard deviation (SD) increase of baseline miRNA expression was calculated as *β*_*1*_ + *β*_*3*_. To facilitate the interpretability of results, we reported these *β*_*1*_ + *β*_*3*_ coefficients after converting them to their original units *post-hoc* (i.e., mm^3^ for volumetric measures, ratio for hippocampal asymmetry). All available brain imaging data at any time point were included in the model and contributed towards the coefficients. As with the cross-sectional analysis, we adjusted the models for age, sex, miRNA batch, and, in the case of hippocampal volume and total brain volume, for eTIV. We additionally calculated the proportion of the variance of change rates explained by statistically significant miRNAs. We created a base mixed-effect model with covariates and random effects as described above, but not including any miRNAs, as well as a full model that additionally included all significant miRNAs and their interactions with age. We then calculated the proportion of the explained variance of the random slope as:

Explained variance (%) = [1- random slope variance (full model)/random slope variance (base model)]*100

For both the cross-sectional and longitudinal analyses, we performed sensitivity analyses by additionally adjusting for baseline blood cell counts (neutrophils, eosinophils, basophils, monocytes, lymphocytes, erythrocytes, nucleated erythrocytes, platelets), blood lipid levels (low-density lipoprotein, high-density lipoprotein, triglycerides), and eGFR. Additionally, we re-ran models after exclusion of participants who, at baseline, had a self-reported physician-diagnosed neurological disorder that could affect hippocampal volume, i.e., dementia (n = 1), Parkinson’s disease (n = 3), multiple sclerosis (n = 10), hippocampal sclerosis (n = 1).

To determine whether age and sex modified the association between miRNAs and imaging measures, we repeated the cross-sectional and longitudinal analyses in age and sex strata. To ensure that the age strata were of equal size so that age-stratified analyses had comparable statistical power, we defined age strata based on the median age at baseline, i.e., age ≥ 54 years and age < 54 years.

The P-values of regression coefficients were corrected for multiple testing using the Benjamini-Hochberg false discovery rate (fdr) method [38] for the number of examined miRNAs (n = 415). The statistical significance threshold was set at 0.05 (fdr ≤ 0.05). For miRNAs recently found to predict Mild Cognitive Impairment (MCI) and its conversion to AD [4], we also considered nominally significant P-values, i.e. without multiple testing adjustment (P value ≤ 0.05).

All analyses were performed in R version 4.1.0.

### MiRNA expression in tissues and cells

To determine in which tissues miRNAs were mostly expressed, we queried the miRNATissueAtlas2 [39]. This online resource contains miRNA sequencing data from samples taken from 60 human tissues across the entire body. We first converted the expression of each miRNA across all tissues into a z-score. Next, for each tissue, we calculated the median of miRNA expression across donors and examined the tissues with the highest median expression. The miRNATissueAtlas2 also included the Tissue Specificity Index. This continuous metric ranges from 0–1. Values closer to 1 indicate that a miRNA is only expressed in a few or a single tissue.

To examine in which cells miRNAs were mostly expressed, we downloaded an atlas based on the Functional Annotation of Mammalian Genome (FANTOM5) project [40]. After exclusion of cancer cell lines and cell lines treated with factors that would alter physiological miRNA expression, 78 unique cell types were included in the analysis. Then, we averaged miRNA expression across multiple samples, and we z-transformed expression across cells for each miRNA to facilitate comparisons.

### Functional genomics and pathway enrichment analysis

To identify potential target genes of miRNAs, we first obtained putative miRNA target genes from MirTarBase (version 9.0) [41], TargetScan (version 8.0) [42] and miRDB (version 6.0) [43]. We then used linear regression, adjusting for age, sex, blood cell counts, miRNA sequencing batch, and gene sequencing batch to identify target genes negatively associated with their targeting miRNA (uncorrected P value ≤ 0.05). We used these genes to perform a pathway enrichment analysis in the Gene Ontology: Biological Processes database [44]. Significantly enriched pathways were determined at the fdr ≤ 0.05 significance threshold. We grouped similar terms with the *rrvgo* (version 1.6.0) R Bioconductor package [45] and further clustered these terms in six broader categories using the *simplifyEnrichment* (version 1.4.0) R Bioconductor package [46] (Supplementary Methods 2).

As an additional analysis, we filtered identified target genes for high expression in the hippocampus. We downloaded RNA consensus tissue gene data from The Human Protein Atlas (version 23.0) [47] and set a cut-off of gene expression > 10 normalized Transcripts per Million in the hippocampal formation.

### MiRNA expression quantitative trait loci (miR-eQTLs) and Mendelian randomization analysis

To identify Single Nucleotide Polymorphisms (SNPs) influencing miRNA expression, we conducted a cross-sectional genome-wide miR-eQTL analysis based on 2456 study participants, for which both genetic and miRNA expression data were available at baseline. We adjusted for age, sex, miRNA batch, and the first ten genetic principal components [32]. Genome-wide significance was defined as P-value ≤ 5 × 10^−8^. We then performed a two-sample Mendelian randomization analysis to examine whether the associations between miRNAs (exposure) and imaging measures (outcome) could be causal. We included as instrumental variables *cis*-miR-eQTLs, defined as those located within 1 MB of miRNA sequences and significant at the P-value ≤ 1 × 10^−5^ threshold, clumped based on linkage disequilibrium (r^2^ < 0.001 within a 10 Mb window) [48]. Depending on whether miRNAs were identified in the cross-sectional or longitudinal analysis, we used summary statistics of published GWAS of left and right hippocampal volume (n = 21 282 participants) [49] or hippocampal atrophy (n = 15 640 participants) [50]. For miRNAs associated with hippocampal volume cross-sectionally, we additionally examined for reverse causation by coding left or right hippocampal volume as the exposure and miRNA expression as the outcome (Supplementary Methods 3).

## Results

### Participant characteristics and descriptive statistics

Study participants were generally well-educated, with a relatively low prevalence of smoking, major cardiovascular, or major neurodegenerative diseases (Table 1**)**. All brain imaging measures were approximately normally distributed both at baseline and during the follow-up examinations (Supplementary Figure S2). At baseline, the right hippocampus was, on average, larger than the left (paired t-test P value < 10^−15^; Table 1). Right and left hippocampal volumes were highly correlated (Pearson’s correlation coefficient: 0.90, 95% CI: 0.89 to 0.90). Hippocampal volume tended to decrease bilaterally with age, especially in older participants (Supplementary Figure S3). Controlling for age and sex, larger hippocampal volume was associated with better global cognition both for the left (standardized beta: 0.05, 95% CI: 0.01 to 0.09, P-value: 0.02) and right (standardized beta: 0.07, 95% CI: 0.03 to 0.11, P-value: 7.94 × 10^−4^) hippocampus. On average, right hippocampal volume decreased faster (mean right hippocampal volume change per year: - 24.75 mm^3^, 95% CI: −79.15 to 29.85 mm^3^; mean left hippocampal volume change per year: −22.52 mm^3^, 95% CI: −74.90 to 29.66 mm^3^; paired t-test P value: 9.12  × 10^−4^). The yearly change rates of left and right hippocampal volume were only moderately correlated (Pearson’s correlation coefficient: 0.56, 95% CI: 0.53 to 0.60).

**Table 1:** Participant characteristics at baseline for each subset of the study sample.

Variable	Subset 1: Baseline participants with complete miRNA data,^*1*^ N = 2928	Subset 2: Baseline participants with complete baseline miRNA and hippocampal MRI data,^*2*^ N = 2062	P value,^*3*^ Comparison of Subsets 1 and 2	Subset 3: Baseline participants with complete baseline miRNA and follow-up hippocampal MRI data,^*4*^ N = 1634	P value,^*3*^ Comparison of Subsets 2 and 3
Sex, n(%)			0.63		0.93
Female	1627 (56%)	1160 (56%)		917 (56%)	
Male	1301 (44%)	902 (44%)		717 (44%)	
Age (years), mean (SD)	55.40 (14.35)	54.48 (14.00)	**0.02**	53.64 (13.13)	0.06
Age range (years)	30.29 – 95.38	30.29 – 95.38		30.29 – 88.50	
Parkinson’s disease, n(%)	11 (0.4%)	4 (0.2%)	0.32	2 (0.1%)	0.60
Dementia, n(%)	4 (0.1%)	1 ( < 0.1%)	0.38	1 ( < 0.1%)	0.87
Multiple Sclerosis, n(%)	13 (0.4%)	10 (0.5%)	0.89	9 (0.6%)	0.80
Uncontrolled hypertension, n(%)	684 (23%)	478 (23%)	0.46	346 (21%)	0.50
Uncontrolled diabetes, n(%)	133 (4.5%)	79 (3.8%)	0.50	48 (2.9%)	0.34
Stroke, n(%)	50 (1.7%)	26 (1.3%)	0.30	25 (1.5%)	0.36
Coronary artery disease	129 (4.4%)	59 (2.9%)	**0.04**	46 (2.8%)	0.45
Educational level, n(%)^*5*^			0.11		0.54
low	60 (2.1%)	35 (1.7%)		21 (1.3%)	
middle	1276 (44%)	850 (42%)		658 (40%)	
high	1565 (54%)	1160 (57%)		946 (58%)	
Current smoking, n(%)	381 (13%)	279 (14%)	0.72	215 (13%)	0.64
Left Hippocampal Volume (mm^3^), mean (SD)	-	3.89 × 10^3^ (0.46 × 10^3^)	-	3.92 × 10^3^ (0.45 × 10^3^)	0.27
Right Hippocampal Volume (mm^3^), mean(SD)	-	4.04 × 10^3^ (0.50 × 10^3^)	-	4.07 × 10^3^ (0.49 × 10^3^)	0.26
Hippocampal asymmetry index, mean(SD)	-	−0.02 (0.03)	-	−0.02 (0.03)	0.32
Total brain volume (mm^3^), mean(SD)	-	1.12 × 10^6^ (0.12 × 10^6^)	-	1.12 × 10^6^ (0.12 × 10^6^)	1.00
Estimated total intracranial volume (mm^3^), mean(SD)	-	1.55 × 10^6^ (0.15 × 10^6^)	-	1.56 × 10^6^ (0.16 × 10^6^)	0.59

^*1*^The subsets used for functional genomics and miR-eQTL analyses were derived from Subset 1, after removing participants with missing gene expression data (n = 565) or genetic data (n = 472), respectively.

^*2*^Data missing for total brain volume (n = 17).

^*3*^Intergroup differences were tested using type III Analysis of Covariance.

^*4*^Data missing for total brain volume (n = 15).

^*5*^For educational level, analysis of covariance was performed for two groups: high education and middle-low education.

Statistically significant differences (*P*-value ≤ 0.05) are shown in bold letters.

### Cross-sectional associations of miRNA expressions with imaging measures

**Fig. 1:**
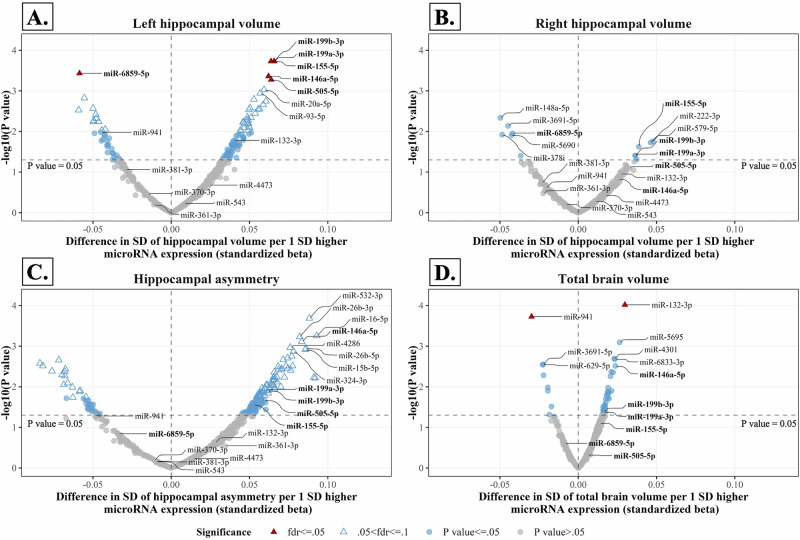
Cross-sectional associations of miRNAs with brain imaging measures. The volcano plots show the association of miRNAs with **A**. left hippocampal volume, **B**. right hippocampal volume, **C**. the hippocampal asymmetry index and **D**. total brain volume. MiRNAs significantly associated with hippocampal volume after FDR correction are displayed in bold. SD Standard Deviation, fdr False Discovery Rate.

Overall, significant baseline associations of miRNA expressions with hippocampal volume were more abundant and markedly stronger for the left hippocampus, while they differed from the associations with total brain volume. Specifically, higher expressions of miR-199a-3p, miR-199b-3p, miR-155-5p, miR-146a-5p, and miR-505-5p were significantly associated with larger left hippocampal volume, while higher expression of miR-6859-5p was significantly associated with smaller left hippocampal volume. While the same miRNAs were among the top results for right hippocampal volume, the effects were much weaker and no associations survived adjustment for multiple testing (Fig. 1 & Supplementary Table S1). As miR-199a-3p and miR-199b-3p have the same mature sequence [51], were very similarly distributed in our data (Supplementary Figure S4), and were similarly associated with hippocampal volume, we treated them as a single cluster (henceforth named miR-199a-3p/miR-199b-3p) for functional and genomics analyses. Beyond that, the miRNAs associated with left hippocampal volume belong in different families and are not clustered in nearby genomic locations.

Interestingly, higher expressions of three miRNAs, miR-125b-5p, miR-18a-5p, and miR-26b-5p, recently found to predict conversion from early MCI to AD [4], were associated with larger left hippocampal volume at nominal significance.

No miRNAs were significantly associated with hippocampal asymmetry (fdr>0.05 for all associations; Fig. 1C**&** Supplementary Table S1). Significant associations with total brain volume were found for miR-132-3p and miR-941, but for none of the miRNAs associated with left hippocampal volume (Fig. 1D**&** Supplementary Table S1).

When additionally controlling for blood cell counts, lipid levels, and eGFR, or removing participants with neurological diseases, associations of miRNAs with all MRI measures remained highly similar (Supplementary Figure S5). When stratifying by age, miRNA associations with left hippocampal volume remained similar and only slightly differed between younger (age < 54 years) and older (age ≥ 54 years) participants **(**Supplementary Figure S6**)**. When we stratified by sex, we observed little difference between men and women for left hippocampal volume, right hippocampal volume, and hippocampal asymmetry. However, four new miRNAs (miR-4473, miR-629-5p, miR-4781-3p, and miR-330-5p) were significantly associated with total brain volume in men only **(**Supplementary Figure S7**)**.

### Associations of baseline miRNA expressions with longitudinal change of imaging measures

**Fig. 2:**
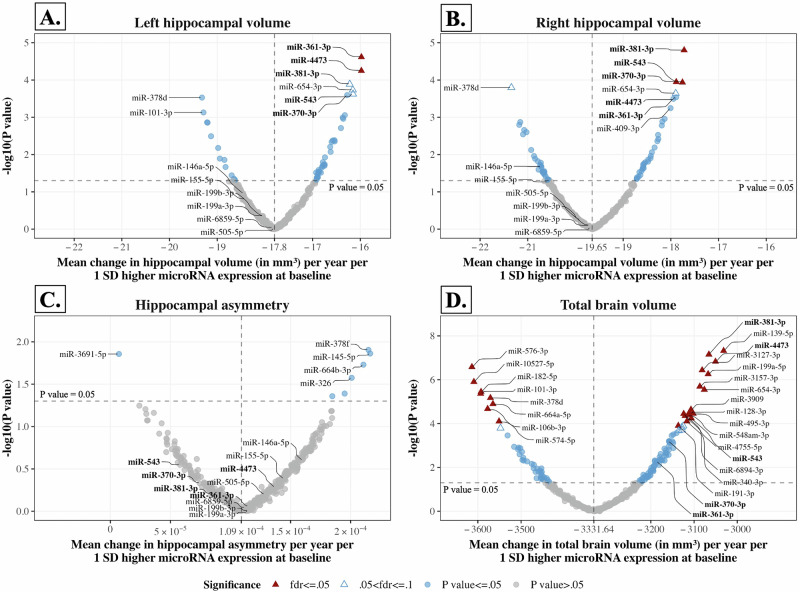
Longitudinal associations of baseline miRNAs with brain imaging measures. The volcano plots show the association of baseline miRNAs with the yearly change rates of **A**. left hippocampal volume, **B**. right hippocampal volume, **C**. hippocampal asymmetry, and **D**. total brain volume. MiRNAs significantly associated with the yearly change of hippocampal volume after FDR correction are displayed in bold. The vertical dashed line indicates the yearly change rate of each measure when miRNA expression was equal to the population mean, averaged across all miRNAs. For miRNAs located at the right side of this line, higher baseline miRNA expression was associated with a slower rate of decline of imaging measures (e.g., slower hippocampal atrophy rate). To facilitate plotting, different axis scales have been used for hippocampal volume, asymmetry, and total brain volume. SD Standard Deviation, fdr False Discovery Rate.

Contrary to the cross-sectional analysis, we observed many similarities in the longitudinal associations of baseline miRNA expressions with the atrophy rates of the left hippocampus, right hippocampus, and total brain. Increased baseline expression of miR-361-3p and miR-4473 was associated with significantly slower left hippocampal atrophy rates over time. For example, in the linear mixed-effect model for miR-361-3p, the average yearly change rate of left hippocampal volume was -17.86 mm^3^ (−0.46% per year compared to baseline). However, when baseline miR-361-3p expression was one SD higher, the average yearly change rate was modified to −15.98 mm^3^ (−0.41% per year compared to baseline), corresponding to a relative decrease of 10.52% of yearly hippocampal atrophy. Similarly, increased baseline expressions of miR-381-3p, miR-370-3p, and miR-543 were associated with significantly slower right hippocampal atrophy rates over time (Fig. 2 and Supplementary Table S2). Notably, these three miRNAs are clustered nearby in the genome at the DLK1-DIO3 genomic region (14q32 locus), but belong to different families [51]. The top miRNAs associated with left and right hippocampal atrophy rates were highly similar (Fig. 2 and Supplementary Table S2). Thus, we considered the abovementioned five miRNAs (miR-361-3p, miR-4473, miR-381-3p, miR-370-3p, and miR-543) as associated with both left and right hippocampal atrophy. Importantly, when included together in a single model, expressions of these miRNAs jointly explained 27.95% and 18.07% of the variance of left and right hippocampal atrophy rates, respectively.

When examining the longitudinal associations of miRNAs with hippocampal asymmetry, we found no significant results. Overall, longitudinal associations were stronger for total brain atrophy than hippocampal atrophy. Baseline expressions of 24 miRNAs were significantly associated with total brain atrophy rate over time. The five miRNAs associated with hippocampal atrophy rate were nominally associated with total brain atrophy rate, while miR-381-3p, miR-4473, and miR-543 survived adjustment for multiple testing (Fig. 2 and Supplementary Table S2).

Again, including blood cell counts, lipid levels, and eGFR in the model or removing participants with neurological diseases at baseline only minimally affected the longitudinal associations of miRNAs with the imaging measures (Supplementary Figure S8).

When stratifying by age, miRNA associations with hippocampal and total brain atrophy rates were similar but tended to be stronger in the younger group. The main exception was miR-361-3p, which had a much stronger association with left hippocampal atrophy rate in the older group. Moreover, in the younger group, higher expressions of four different miRNAs were significantly associated with left and right hippocampal atrophy rates (left: miR-101-3p, miR-106b-3p, right: miR-101-3p, miR-664a-5p, miR-3157-3p; Supplementary Figure S9**)**. Sex stratification showed that miRNA associations with left and right hippocampal atrophy rates were generally similar in men and women. However, one miRNA, miR-125b-5p, was significantly associated with faster hippocampal atrophy rate in women only **(**Supplementary Figure S10**)**. This miRNA was among those recently found to predict conversion from early MCI to AD [4]. Moreover, it was also associated with faster left and right hippocampal atrophy rates at a nominally significant threshold in our entire cohort (Supplementary Table S2**)**.

### MiRNA expression in tissues and cells

**Fig. 3:**
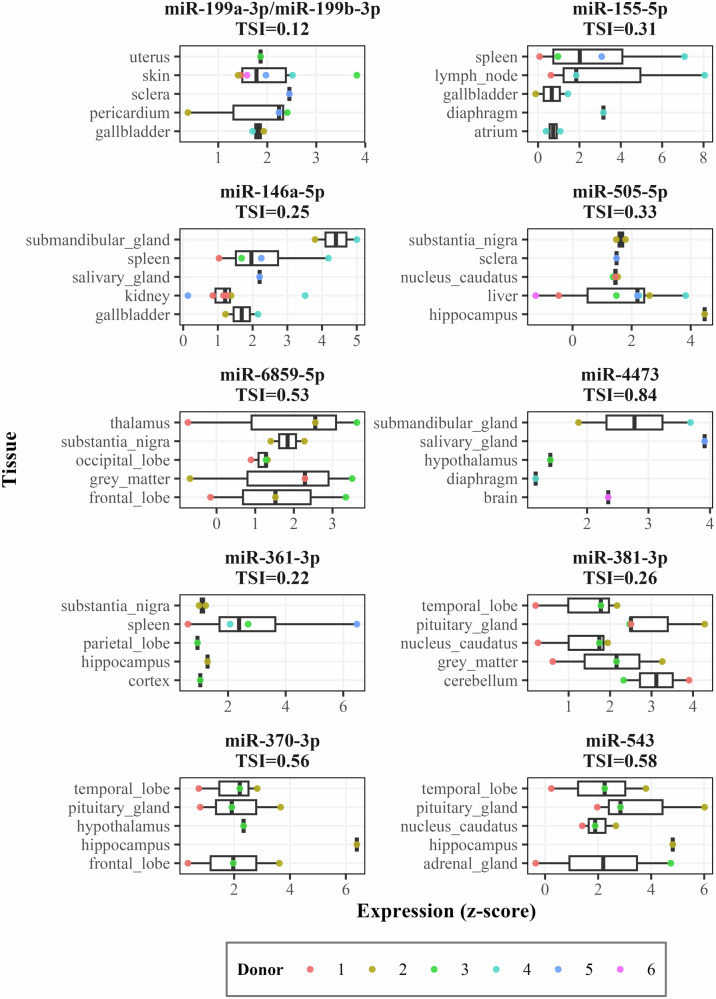
Tissue expression analysis of miRNAs associated with hippocampal volume cross-sectionally or longitudinally. Data was obtained from the miRNATissueAtlas [39], based on post-mortem samples taken from 6 human donors (here indicated by the colored dots). The boxplots show the top 5 tissues for each miRNA, based on median miRNA expression. Note that, to allow for better comparison of relative expression in tissues for each miRNA, the scale of the x-axis (expression z-score) varies. TSI Tissue Specificity Index.

Next, we examined the tissue expression of the miRNAs we identified in the cross-sectional and longitudinal analyses. We determined a high expression in brain tissues for miR-505-5p and miR-6859-5p, from the cross-sectional analysis, as well as miR-361-3p, miR-4473, miR-381-3p, miR-543, and miR-370-3p from the longitudinal analysis. Moreover, miR-4473 was highly tissue-specific (Tissue Specificity Index: 0.83; Fig. 3) and was highly expressed in neuronal cells (Supplementary Figure S11).

### Functional genomics and pathway enrichment analysis

**Fig. 4:**
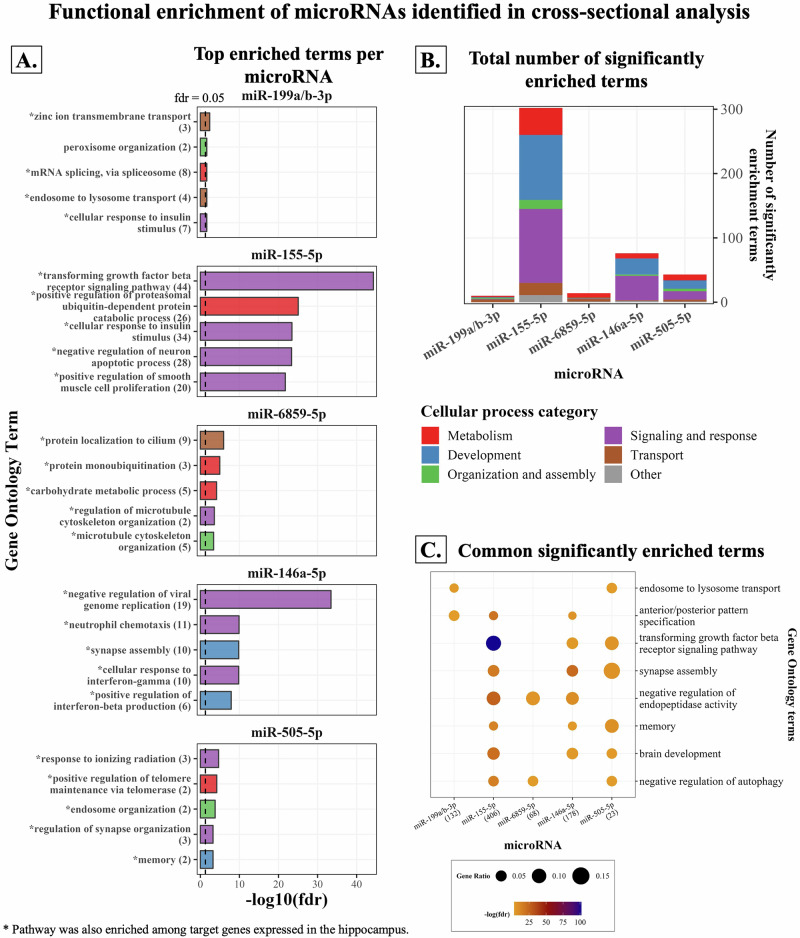
Results of *Gene ontology: Biological Process* enrichment analysis for target genes of miRNAs identified in the cross-sectional analysis. MiRNA target genes were determined based on in silico prediction and a negative association between miRNA and gene expressions, as measured in blood samples. The top five significantly enriched pathways (lowest p-value) are shown in the bar plots in **A**., with the number of target genes belonging to each pathway in parentheses. Pathways marked with an asterisk (*) were also enriched when filtering for target genes with high expression in the hippocampus, based on data from The Human Protein Atlas [47]. Significantly enriched pathways for each miRNA were grouped in broad categories, indicated by bar colors. The total number of significantly enriched pathways for each miRNA is shown in **B**. Pathways enriched across the target genes of multiple ( ≥ 3) miRNAs are shown in the bubble plot in **C**. Numbers in parentheses next to miRNA names indicate the total number of target genes of this miRNA that was included in *Gene Ontology*. P-values have been adjusted for multiple testing using the Benjamini-Hochberg false discovery rate method. fdr False Discovery Rate.

**Fig. 5:**
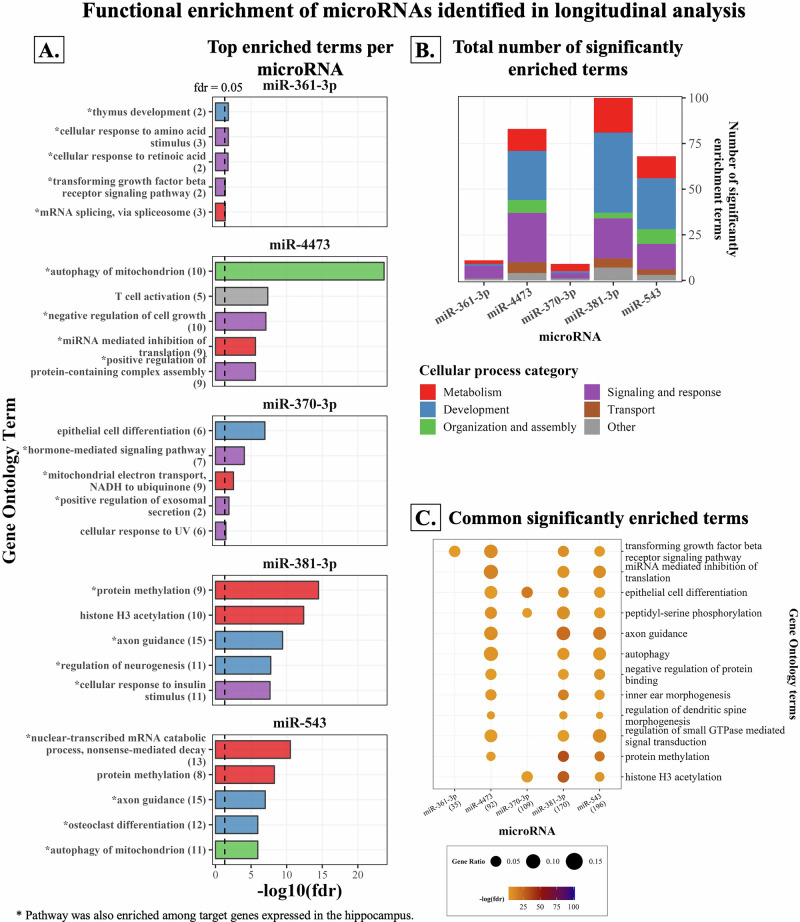
Results of *Gene ontology: Biological Process* enrichment analysis for target genes of miRNAs identified in the longitudinal analysis. MiRNA target genes were determined based on in silico prediction and a negative association between miRNA and gene expressions, as measured in blood samples. The top five significantly enriched pathways (lowest p-value) are shown in the bar plots in **A**., with the number of target genes belonging to each pathway in parentheses. Pathways marked with an asterisk (*) were also enriched when filtering for target genes with high expression in the hippocampus, based on data from The Human Protein Atlas [47]. Significantly enriched pathways for each miRNA were grouped in broad categories, indicated by bar colors. The total number of significantly enriched pathways for each miRNA is shown in **B**. Pathways enriched across the target genes of multiple ( ≥ 3) miRNAs are shown in the bubble plot **C**. Numbers in parentheses next to miRNA names indicate the total number of target genes of this miRNA that was included in *Gene Ontology*. P-values have been adjusted for multiple testing using the Benjamini-Hochberg false discovery rate method. fdr False Discovery Rate.

Functional genomics analysis determined that the miRNAs identified in the cross-sectional and the longitudinal analysis had 817 and 586 unique potential target genes, respectively **(**Supplementary Figure S12A**&** Supplementary Table S3**)**. Generally, the identified miRNAs had relatively few common target genes (Supplementary Figure S12B**)**. Gene ontology enrichment of the target genes of each miRNA highlighted putative miRNA-regulated pathways. For example, miR-146a-5p targeted genes related to viral defense and inflammation, miR-6859-5p targeted genes related to cytoskeleton organization, and miR-4473 targeted genes related to mitochondrial autophagy and cell growth. Overall, the majority of target genes of identified miRNAs were related to cellular development, signaling, and response to stimuli (Figs. 4, 5**&** Supplementary Table S4).

Νotably, “transforming growth factor beta (TGF-β) receptor signaling” pathway was significantly enriched among targets of three miRNAs identified in the cross-sectional analysis (miR-155-5p, miR-146a-5p, miR-505-5p) and four miRNAs identified in the longitudinal analysis (miR-361-3p, miR-4473, miR-381-3p, and miR-543; Fig. 5C & Supplementary Table S4), Moreover, the “memory”, “brain development”, and “synapse assembly” pathways were each enriched for three of the miRNAs identified in the cross-sectional analysis (Fig. 4C**)**. The “axon guidance” and “dendritic spine morphogenesis” pathways were enriched for three of the miRNAs identified in the longitudinal analysis (Fig. 5C**)**. Lastly, miR-155-5p and miR-381-3p targeted genes related to amyloid-beta formation and clearance, including *PSEN1* for miR-155-5p (Supplementary Table S4).

We found high expressions in the hippocampus for 460 of the 822 (55.96%) target genes of miRNAs identified in the cross-sectional analysis and 329 of the 587 (56.05%) target genes of miRNAs identified in the longitudinal analysis. Most of the top enriched pathways identified in the main analysis were also enriched for hippocampus-specific genes (Figs. 4, 5 & Supplementary Figure S13).

### miR-eQTL and Mendelian randomization analysis

Among the miRNAs from the longitudinal analysis, we identified in total 19 unique independent lead genome-wide significant (P value ≤ 5 × 10^−8^) *cis-*SNPs that were associated with the expressions of either miR-370-3p, miR-381-3p, miR-543, or miR-4473. Among the miRNAs identified in the cross-sectional analysis, 10 unique lead independent genome-wide significant *trans-*SNPs but no *cis*-SNPs were associated with the expressions of miR-146a-5p or miR-6859-5p **(**Supplementary Table S5**)**. We did not perform the miR-eQTL analysis for miR-505-5p and miR-361-3p, which are located on the X chromosome, for which no genotype data were available.

After clumping of all SNPs that reached suggestive significance (P value ≤ 1 × 10^−5^), 7 independent *cis*-miR-eQTLs remained as genetic proxies for miR-146a-5p from the cross-sectional analysis and miR-370-3p, miR-381-3p, miR-543, and miR-4473 from the longitudinal analysis. Using these SNPs as genetic instruments, we performed two-sample Mendelian randomization analyses to determine potential causal effects of miR-146a-5p on left hippocampal volume and right hippocampal volume, as well as miR-381-3p, miR-543, miR-370-3p, and miR-4473 on hippocampal atrophy rate. We found no evidence of a causal influence of these miRNAs on hippocampal volume. However, when assessing for reverse causation, we found evidence for a causal association of larger left and right hippocampal volume with lower miR-6859-5p expression. The F-statistic for all Mendelian randomization analyses ranged between 24.32 and 139.31, indicating a low chance of weak instrument bias **(**Supplementary Table S6**)**.

## Discussion

In the present study, we identified six miRNAs that were cross-sectionally associated with left hippocampal volume only. A different signature of five miRNAs was not only associated with hippocampal atrophy rate bilaterally, but also with total brain atrophy rate. The difference in the results of the cross-sectional and longitudinal analyses suggests involvement of different miRNAs in hippocampal early-life development and aging. To further disentangle the biogenesis and function of identified miRNAs, we used genomics and transcriptomics data. Indeed, we found that many of them were genetically influenced and were associated with target genes related to brain development, memory, synapse assembly, axon guidance, and dendrite morphogenesis. Moreover, some were primarily expressed in brain tissue, albeit with low tissue specificity.

Expressions of miR-199a-3p/miR-199b-3p, miR-155-5p, miR-146a-5p, miR-6859-5p, and miR-505-5p were cross-sectionally associated with left hippocampal volume, but not right hippocampal or total brain volumes. These distinct association patterns could indicate that these miRNAs are linked with lateralized hippocampal development [52]. Our enrichment analysis supports such an involvement in development for miR-155-5p, miR-146a-5p, and miR-505-5p, which targeted genes enriched for brain development, axon guidance, and TFG-β signaling, a pathway crucial for brain development and function [53]. This requires further experimental validation, as our analysis of target genes was based on blood-derived miRNA and gene expression data. For miR-199a-3p/miR-199b-3p, involvement in hippocampal development is particularly supported by previous studies, as these miRNAs have been implicated in dendrite development [54], early life neurogenesis, and neuronal differentiation [55, 56].

Beyond hippocampal development, the six miRNAs associated with left hippocampal volume cross-sectionally could be involved in processes affecting hippocampal volume later in life, such as aging or neurodegeneration. Indeed, miR-146a-5p and miR-155-5p are well known for their roles in neuroinflammation [57, 58], hippocampal neurotoxicity [59, 60], and AD pathology [61, 62]. Moreover, in a previous study based on the same dataset, we found that miR-146a-5p and miR-155-5p belong in the same co-expression cluster, suggesting they might have similar biological functions [32]. Despite their putative neurotoxic role, these two miRNAs were associated with a larger hippocampus in our study. Although a larger cross-sectionally measured hippocampus is generally considered as indicative of better brain health, this is not always the case [23]. For example, given the involvement of miR-146a-5p and miR-155-5p in neuroinflammation, their association with a larger hippocampus could reflect disruptions in blood-brain-barrier permeability and cerebral edema in neuroinflammatory states [63, 64]. The discrepancy could also be because our miRNAs were measured in peripheral blood and could reflect systemic rather than brain-specific processes, especially given the low tissue specificity of miR-155-5p and miR-146a-5p, as well as their high expression in tissues outside the brain and especially in lymphocytes. Despite this uncertainty regarding the mechanistic involvement of these miRNAs in brain function, our results, combined with their known role in AD, suggest them as good candidates for its early detection. This applies especially to miR-146a-5p, which was found by two previous meta-analyses to be downregulated in the blood [65] and cerebrospinal fluid [5] of AD patients, but upregulated in their brains [65]. In addition to AD, miR-146a-5p has also been suggested as an early biomarker of age-related cognitive dysfunction [66] and has been linked to cognitive performance in the general population [67].

Mendelian randomization analyses did not detect causal links between identified miRNAs and any of our cross-sectional or longitudinal hippocampus measures. However, the lack of significant results in Mendelian randomization analyses is not sufficient evidence to exclude causality [68]. Our analysis was limited by having few to no identified genetic instruments for examined miRNAs, due to the relatively small sample used for miRNA-eQTL analysis. Moreover, investigations for reverse causation returned no significant results, except for miR-6859-5p. Mendelian randomization indicated that a larger hippocampus leads to lower expression of this miRNA. Our enrichment analysis suggests that this little-studied miRNA is involved in essential cellular functions like cytoskeleton organization. Thus, larger hippocampi with more or larger cells might require larger amounts of miR-6859-5p to maintain neurogenesis and axonogenesis [69], leading to its reduced secretion in the periphery [70]. As the function of this miRNA is not well known, this hypothesis should be tested by future experimental studies to provide further insight into its potential role in the brain.

In contrast to the cross-sectional analysis, a common signature consisting of miR-361-3p, miR-4473, miR-381-3p, miR-543, and miR-370-3p, measured at study baseline, was similarly associated with the longitudinal atrophy rates of the left hippocampus, right hippocampus, and whole brain. Despite their low tissue specificity, these miRNAs were most highly expressed in brain tissue compared to the rest of the body. Notably, miR-370-3p, miR-381-3p, and miR-543 are all located within the DLK1-DIO3 genomic region. This region contains the largest cluster of miRNAs in the genome, many of which perform critical neuronal functions [71]. Moreover, we previously found that miR-370-3p, miR-361-3p, and miR-543 were nominally associated with cognitive domains (executive function, working memory, crystalized intelligence), although the associations did not survive adjustment for multiple testing, while miR-370-3p and miR-381-3p belonged to the same co-expression cluster [32]. Previous studies have also linked miR-361-3p, miR-370-3p, and miR-381-3p to neurodegeneration. Specifically, miR-361-3p protected against AD-related neuronal injury and cognitive deficits [72], miR-370-3p impaired hippocampal neurogenesis [73], while miR-381-3p attenuated neurotoxicity induced by Αβ [74] and ischemia [75]. Importantly, in meta-analyses, miR-370-3p tended to be downregulated in the brain [65] and miR-381-3p was upregulated in the cerebrospinal fluid [5] of AD patients. The involvement of these miRNAs in AD, their high brain expression levels, as well as their association with hippocampal atrophy and cognition in our relatively young and healthy cohort, mark them as ideal candidates for the early detection of AD. Given that early AD preferentially targets the hippocampus [16], this applies especially to miR-370-3p, which was much more strongly associated with hippocampal rather than total brain atrophy rate. Contrary to miR-370-3p, miR-4473 was strongly associated with both hippocampal and total brain atrophy rates. In addition, it was cross-sectionally associated with total brain volume in men and was the only identified miRNA that was with high specificity expressed in brain tissue and neuronal cells. Future studies could explore the potential of this little-studied miRNA as a novel, easily accessible marker of unhealthy brain aging.

A recent study identified miR-125b-5p, miR-18a-5p, and miR-26b-5p as the best predictors of conversion from early MCI to AD [4]. We found that these three miRNAs were cross-sectionally associated with left hippocampal volume at nominal significance. Additionally, miR-125b-5p was longitudinally associated with hippocampal atrophy rate, especially in women, and has been associated with working memory in a previous study by our group [32]. These overlapping results suggest that these three miRNAs, and especially miR-125b-5p, are involved in early AD in a sex-specific manner and could be used for its detection.

As mentioned, miR-146a-5p, miR-370-3p, miR-361-3p, miR-543, miR-381-3p, miR-125b-5p, miR-18a-5p, and miR-26b-5p have previously been linked to cognitive performance or AD, extending their association with hippocampal volume or atrophy to a functional outcome. This was not the case for the other hippocampus-related miRNAs, potentially because they do not reflect brain-specific mechanisms. Alternatively, some do reflect mechanisms of aging and neurodegeneration, albeit at an early stage, when brain atrophy has occurred before the ensuing cognitive decline. Further mechanistic insights could be provided by studies examining the association of miRNAs with longitudinal cognitive changes. One such study found that miR-199a-3p/miR-199b-3p, miR-146a-5p, miR-155-5p, miR-505-5p, miR-381-3p, and miR-543 are linked to cognitive trajectories [76]. However, miRNAs were measured in post-mortem brain tissue from a population considerably older (mean age: 81.7 years) than ours (mean age in our study: 55.40 years), limiting the comparability with our results. Thus, further future longitudinal studies investigating the relation of miRNAs to cognition are warranted.

Our study has considerable strengths. First, it was based on a large cohort with a wide age range, increasing precision and statistical power. Second, using next-generation sequencing allowed the untargeted measurement of miRNAs and mRNA transcripts. Lastly, we characterized the biogenesis and functions of identified miRNAs through a multi-omics analysis. Our study also has potential limitations. A major limitation is the lack of replication of our findings. As is common for cohort studies, our single-center study recruited from a predefined geographic area, potentially limiting generalizability. Replication of our findings in other population-based studies is essential to firmly establish the relations of miRNAs with hippocampal volume and atrophy. Such studies would benefit from larger sample sizes and standardization of data collection methods, which would improve comparability and potentially expand our findings. Moreover, we used whole blood samples for RNA sequencing, so that measured miRNAs and mRNAs might have originated from the blood or other peripheral tissues. We accounted for this by controlling for blood cell and lipid levels. Moreover, we used publicly available data to determine the expression levels of identified miRNAs across tissues and cells, with the rationale that brain- and neuron-enriched miRNAs might be more plausibly involved in brain biology. However, it remains probable that the associations of miRNAs with brain volumes observed here reflect systemic processes, especially for miRNAs with low expression in the brain, such as miR-146a-5p, miR-155-5p, and miR-199a-3p/miR-199b-3p. Similarly, the putative miRNA-mediated regulation of target genes and biological pathways in our functional genomics analysis occurred in peripheral blood and does not necessarily reflect brain processes. To account for this, we filtered for target genes highly expressed in the hippocampus. However, our identified miRNA-gene interactions and affected pathways should be viewed as hypothesis-generating and necessitate experimental validation in brain tissue. Lastly, given our use of structural imaging data, it was also not possible to discern if decreases in hippocampal and total brain volume are due to neurodegenerative pathology. Our results might reflect normal aging and their relevance for neurodegeneration should be interpreted in conjunction with past or future experimental studies.

In conclusion, here we identified two different blood-derived miRNA signatures associated with hippocampal volume and its rate of atrophy. Among these miRNAs, miR-146a-5p, miR-370, and miR-125b-5p in particular have been suggested as biomarkers of AD and are good candidates for its presymptomatic detection. Moreover, our study identified novel associations of the little-studied miR-6859-5p and miR-4473 with hippocampal volume and atrophy rate. The effects of these miRNAs on brain function and their relation to neurodegeneration require further investigation. Importantly, our results also need to be replicated by other population-based studies. Lastly, our study identified genetic variants influencing the expression of identified miRNAs, as well as thousands of miRNA-target gene interactions and related biological pathways. These findings provide new testable hypotheses for future experimental studies, which could lead to the development of miRNA-based therapeutics against age-related hippocampal atrophy or neurodegeneration.

## Supplementary information

Supplementary Materials

Supplementary Tables

## Data Availability

The Rhineland Study’s dataset is not publicly available because of data protection regulations. Access to data can be provided to scientists in accordance with the Rhineland Study’s Data Use and Access Policy. Requests for further information or to access the Rhineland Study’s dataset should be directed to RS-DUAC@dzne.de.

## Code availability

The R code used to carry out analyses can be made available upon reasonable request by contacting the authors.
