# Supplementary material for: Blood-derived microRNA signatures associated with hippocampal structure and atrophy rate: findings from the Rhineland Study
Source: Mol Psychiatry. 2026 Apr 24;31(9):5215–27. doi: 10.1038/s41380-026-03611-6 (PMC13441870; doi:10.1038/s41380-026-03611-6)
Supplement: Supplementary file 1 [file 41380_2026_3611_MOESM1_ESM.pdf]

Blood-derived microRNA signatures associated with hippocampal structure and atrophy rate: Findings from the Rhineland Study

Authors
Konstantinos Melas, MD[1], Valentina Talevi, MSc, PhD[1], Mohammed Aslam Imtiaz, MSc, PhD[1], Dennis Krüger, PhD[2], Tonatiuh Pena-Centeno, PhD[2,3], André Fischer, PhD[2,4,5], N. Ahmad Aziz, MD, PhD[1,6,7], Monique MB Breteler, MD, PhD[1,7]

Affiliations
[1] Population Health Sciences, German Centre for Neurodegenerative Diseases (DZNE), Bonn, Germany
[2] Department for Epigenetics and Systems Medicine in Neurodegenerative Diseases (DZNE), German Center for Neurodegenerative Diseases, Göttingen, Germany
[3] Bioinformatics Unit, German Centre for Neurodegenerative Diseases (DZNE), Göttingen, Germany
[4] Department for Psychiatry. and Psychotherapy, University Medical Center Göttingen, Göttingen, Germany
[5] Cluster of Excellence MBExC, University of Göttingen & University Medical Center Goettingen, Göttingen, Germany
[6] Department of Neurology, Faculty of Medicine, University of Bonn, Bonn, Germany
[7] Institute for Medical Biometry, Informatics and Epidemiology (IMBIE), Faculty of Medicine, University of Bonn, Bonn, Germany

Supplementary Materials

Supplementary methods 1-3
Supplementary Figure S1: Study analytical sample and subsets
Supplementary Figure S3: Distribution of brain imaging measures
Supplementary Figure S3: Longitudinal change of brain imaging measures
Supplementary Figure S4: Distribution of normalized counts of miRNAs associated with hippocampal volume
Supplementary Figure S5: Sensitivity analyses of the cross-sectional association of miRNAs with brain imaging measures
Supplementary Figure S6: Age stratification, cross-sectional analysis
Supplementary Figure S7: Sex stratification, cross-sectional analysis
Supplementary Figure S8: Sensitivity analyses of the longitudinal association of miRNAs with brain imaging measures
Supplementary Figure S9: Age stratification, longitudinal analysis
Supplementary Figure S10: Sex stratification, longitudinal analysis
Supplementary Figure S11: MiRNA expression in cells.
Supplementary Figure S12: MiRNA target genes identified through functional genomics
Supplementary Figure S13: Enrichment analysis of miRNA target genes expressed in the hippocampus.
Supplementary Figure S14: Clustering of *Gene Ontology: Biological Processes* with the *SimplifyEnrichment* package.
References

Supplementary methods 1

Data collection for major cardiovascular and neurodegenerative diseases, global cognition, education, smoking, blood cell counts, blood lipid levels, and estimated Glomerular Filtration Rate (eGFR)

The existence of a physician diagnosis of dementia, Parkinson's disease, multiple sclerosis, untreated hypertension, untreated diabetes, stroke, and coronary artery disease was determined based on participant self-reports. Participants were classified as having untreated hypertension when they reported no regular use of antihypertensive medication and either reported having a diagnosis of hypertension, had a mean systolic blood pressure exceeding 140 mmHg, or had a mean diastolic blood pressure exceeding 90 mmHg. Systolic and diastolic blood pressure were measured in a sitting position, using an oscillometric blood pressure device (Omron 705 IT) (1). The measurements were performed thrice, separated by ten minutes, by experienced study technicians while participants were sitting in a resting chair in a quiet environment. The mean of the second and third measurements was used for analysis. Participants were classified as having untreated diabetes when they reported no use of antidiabetic medication and either reported having a diagnosis of diabetes, had a glycated hemoglobin percentage exceeding 6.5%, or had glucose exceeding 126 mg/dL in fasting morning blood.

Global cognition was measured based on an extensive neuropsychological battery measuring fluid cognitive performance across four domains: working memory, episodic verbal memory, processing speed, and executive function (2). Working memory was assessed with the orally performed Digit Span forward and backward task, and the touchpad-based Corsi block-tapping test, based on the Psychology Experiment Building Language battery (3). Episodic verbal memory was evaluated with the Auditory Verbal Learning and Memory test with a list length of 15 words (4). Assessment of processing speed was based on a numbers-only trail-making test (Trail-making test A), and prosaccade latency (time needed to initiate a saccade), derived from an eye movement test battery (5). We examined executive function using the antisaccade error rate (percentage of trials in which the participant made a direction error) from the same battery, combined with a categorical word fluency task (animals), and a number-and-letters trail-making test (Trail-making test B). Cognitive domain performance scores were created by averaging the z-scores of the tests contributing to each domain. The creation of these z-scores was based on data from the first 5000 participants of the Rhineland Study. We averaged the z-scores for working memory, episodic verbal memory, processing speed, and executive function to create the global cognition composite score. All examinations were administered in the German language, following a standardized procedure by certified study technicians.

Information on education and current smoking was based on self-reports. Education was classified as "low" (early childhood education up to lower secondary education), "middle" (secondary education up to Bachelor's or equivalent level), or "high" (Master's or equivalent level up to Doctoral or equivalent level). Participants with missing self-reported data on smoking and with a cotinine level exceeding the non-smoker sample-defined 97.5 percentile were classified as current smokers.

In a sensitivity analysis, complementary to our main analyses, we also included as covariates in linear regression models blood cell counts (neutrophils, eosinophils, basophils, monocytes, lymphocytes, erythrocytes, nucleated erythrocytes, and platelets), blood lipid levels (low-

density lipoprotein, high-density lipoprotein, triglycerides), and eGFR. Blood samples were stored in ethylenediaminetetraacetic acid (EDTA) whole blood tubes for measurement of blood cell counts and in serum tubes with a clotting activator for measurement of biochemical variables. Blood cell counts were measured at the Central Laboratory of the University Hospital Bonn, on a hematological analyzer (Sysmex XN9000). Fasting levels of serum blood lipid levels and cystatin-C were measured on the day of blood withdrawal according to standard procedures at the University Hospital Bonn. We calculated eGFR based on cystatin-C levels using the Chronic Kidney Disease Epidemiology Collaboration (CKD-EPI) formula.

Supplementary methods 2

Functional genomics analysis

For microRNAs (miRNAs) associated with imaging measures, we employed a functional genomics analysis to identify potential miRNA target genes. First, we used the *multimir* R package (version 2.4.0) (6) to obtain a list of putative target genes for each miRNA, from 3 online databases: the MirTarBase (version 9.0) (7) database was inquired for experimentally validated miRNA-target gene interactions, and the TargetScan (version 8.0) (8) and miRDB (version 6.0) (9,10) databases were inquired for predicted miRNA-target gene interactions. Each miRNA-target gene interaction obtained through the *multimir* package is accompanied by a prediction score, which is a measure of prediction confidence and is provided by the inquired databases. To identify all putative miRNA-target genes, we included all predicted target genes, regardless of the prediction score. Moreover, we employed the union of the target genes obtained from the 3 databases to minimize false negatives (11). Subsequently, we determined the association between miRNA expression and expression of their putative target genes using individual-level miRNA and gene expression data available from 2363 participants of our study. We ran separate regression models for each miRNA (independent variable) and each of its putative target genes (dependent variable), adjusting for age, sex, blood cell counts, miRNA sequencing batch, and gene sequencing batch. Lastly, we filtered for target genes that were negatively associated with their targeting miRNA (linear regression beta coefficient ≤ 0 and P value ≤ 0.05). P values for this analysis were not adjusted for multiple testing, as the analysis was performed only for genes putatively targeted by miRNAs, providing an *a priori* hypothesis that they might be associated.

Pathway enrichment analysis

For the miRNA target genes identified in the previous steps, we performed a pathway enrichment analysis using the *clusterProfiler* (version 3.18.1) R Bioconductor package (12,13). We applied an overrepresentation analysis in enrichment terms obtained from the Gene Ontology: Biological Processes (Gene Ontology) database (14,15). The universe (background genes) of the overrepresentation analysis was set to the genes measured in our study. Statistical significance was determined after adjusting for multiple testing with the false discovery rate (fdr) method and at a threshold of fdr ≤ 0.05.

Subsequently, redundant Gene Ontology terms were grouped using the *rrvgo* (version 1.6.0) R Bioconductor package with default parameters (16). Specifically, a semantic similarity matrix of terms was constructed, and similar terms were grouped, selecting the largest term (as defined by the number of genes in each enriched term) to represent each group. A

relatively small similarity threshold of 0.6 was selected, so that only highly redundant terms were grouped. The P values of the terms within each group were pooled using the Fisher method, and multiple testing adjustment with the BH method was performed for the pooled P values. To determine the broader cellular processes that the enriched terms belong to, we further clustered them into six large categories. This was done using the *simplifyEnrichment* (version 1.4.0) R Bioconductor package (17). Specifically, we created a similarity matrix of terms based on gene overlap. We then used the *simplifyGo* function with default parameters to cluster terms based on the similarity matrix, setting the minimal number of terms within a cluster to 7. Resulting clusters were then named based on the most common terms, after visual inspection of word clouds (**Supplementary Figure S14**).

Supplementary methods 3

miR-eQTL analysis

To identify miRNA expression quantitative trait loci (miR-eQTLs), we conducted a genome-wide miR-eQTL analysis on 2456 participants, for which both genetic and miRNA expression data were available. We evaluated the association between each SNP (independent variable) and expression levels of each miRNA (dependent variable) using multivariable linear regression, adjusted for age, sex, miRNA batch, and the first ten genetic principal components. *Cis*-SNPs were defined as those located within 1 Mb (1 Mb before the start or 1 Mb after the end) of the mature miRNA genomic sequence (18). The genome-wide significance level for *cis* miR-eQTLs was set at P value $\leq 5\times10^{-8}$. We used the Functional Mapping and Annotation (FUMA) for GWAS platform (19) to define genomic risk loci by clumping SNPs in linkage disequilibrium at $r^2 > 0.6$. Significant SNPs in relatively high linkage disequilibrium at $r^2 < 0.6$ were defined as independent significant SNPs, while significant SNPs in approximate linkage disequilibrium at $r^2 < 0.1$ were defined as lead SNPs. We mapped genes to lead SNPs with positional mapping.

Two-sample Mendelian Randomization

We employed a Two-sample Mendelian Randomization analysis to examine whether the cross-sectional or longitudinal associations between miRNAs and imaging measures could be causal, using the TwoSampleMR R package (20). We included as instrumental variables *cis*-SNPs that were significant at the suggestive P value $\leq 1\times10^{-5}$ significance level in the miR-eQTL analysis (18) and miRNA expression as the exposure. Depending on whether miRNAs were identified in the cross-sectional or longitudinal analysis, we used as outcome hippocampal volume or atrophy based on published summary statistics from recent genome-wide association studies (GWAS) (21,22). Before the analysis, we clumped SNPs in linkage disequilibrium, defined as $r^2 < 0.001$ within a 10 Mb window. We performed the Mendelian Randomization analysis using the Wald ratio test when only one SNP instrumental variable was available, the inverse variance weighted method, when two SNP instrumental variables were available, and the inverse variance weighted, MR Egger, simple mode, weighted mode, and weighted median methods, when three or more SNP instrumental variables were available. We additionally examined for inverse causation by performing the same analysis, but this time, coding hippocampal volume or atrophy as the exposure and miRNA expression as the outcome. To assess the risk of weak instrument bias, we calculated the F-statistic for the selected SNP instrumental variables.

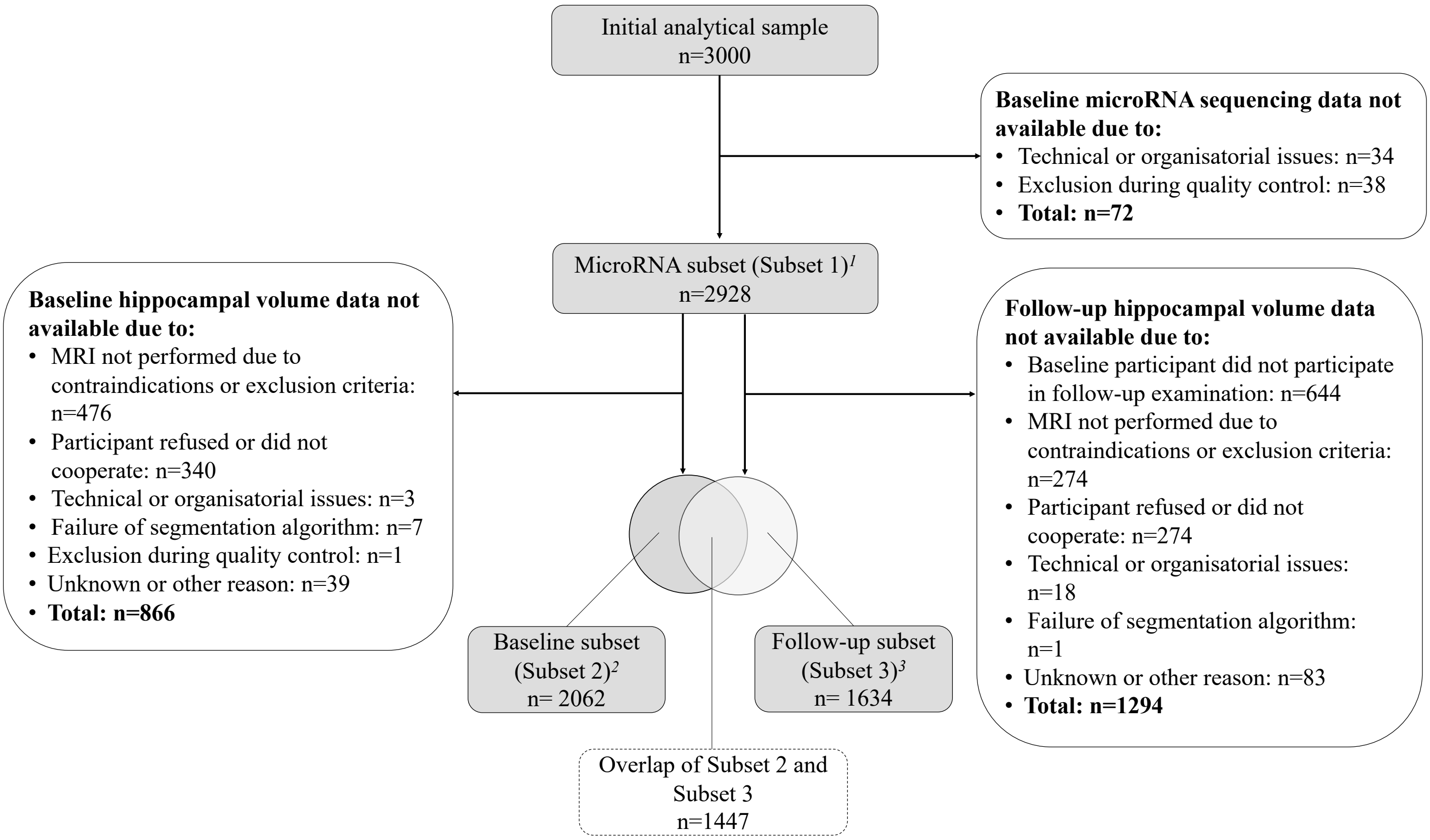

Supplementary Figure S1: Study analytical sample and subsets

[1] The subsets used for functional genomics and miR-eQTL analyses were derived from Subset 1, after removing participants with missing gene expression data (n=565) or genetic data (n=472), respectively.

[2] Data additionally missing for total brain volume: n=17.

[3] Data additionally missing for total brain volume: n=15.

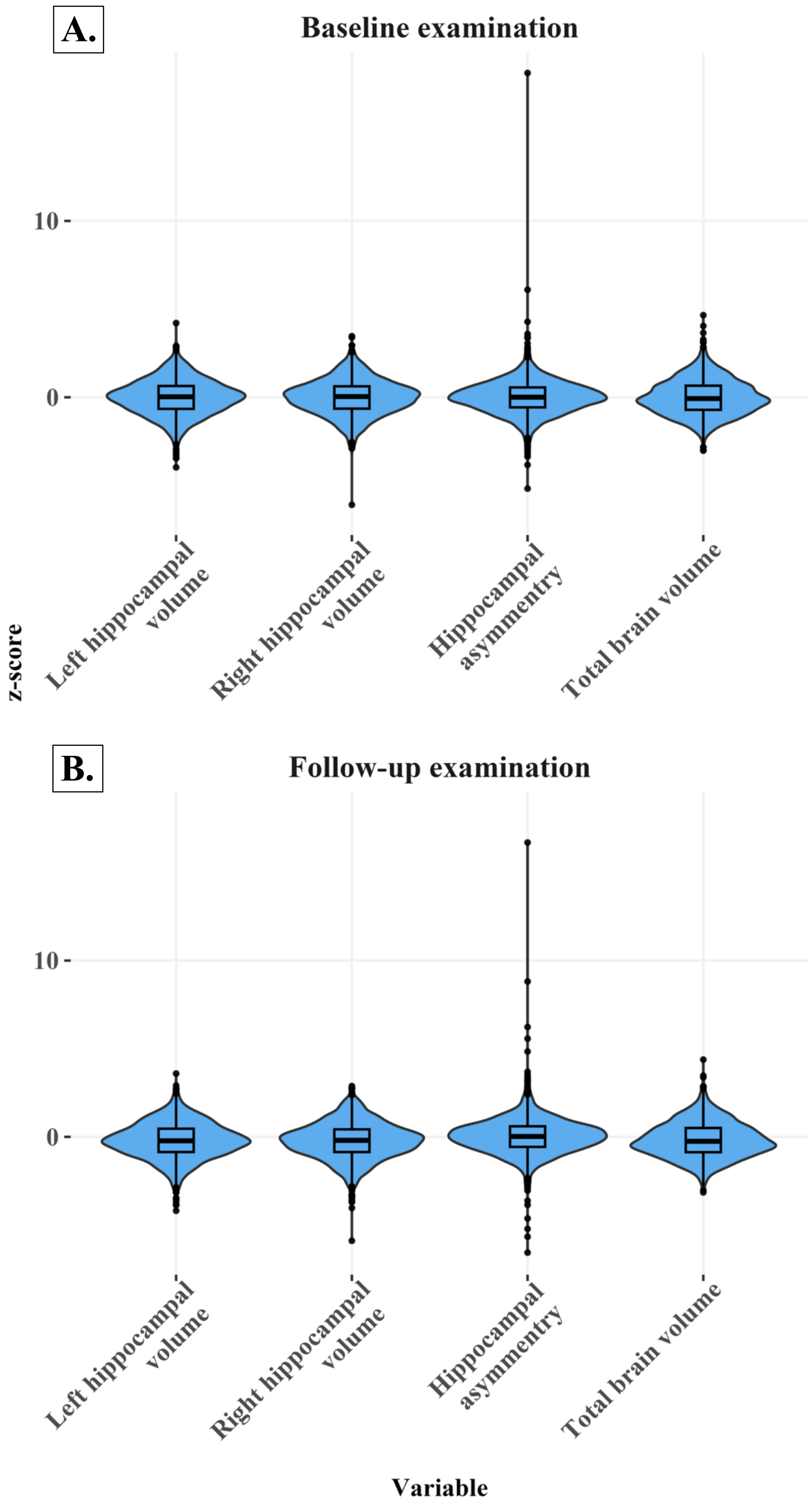

Supplementary Figure S2: Distribution of brain imaging measures
The violin boxplots show the distributions of brain imaging measures at study baseline (**A.**) and at the follow-up examination (**B.**), after z-standardization.

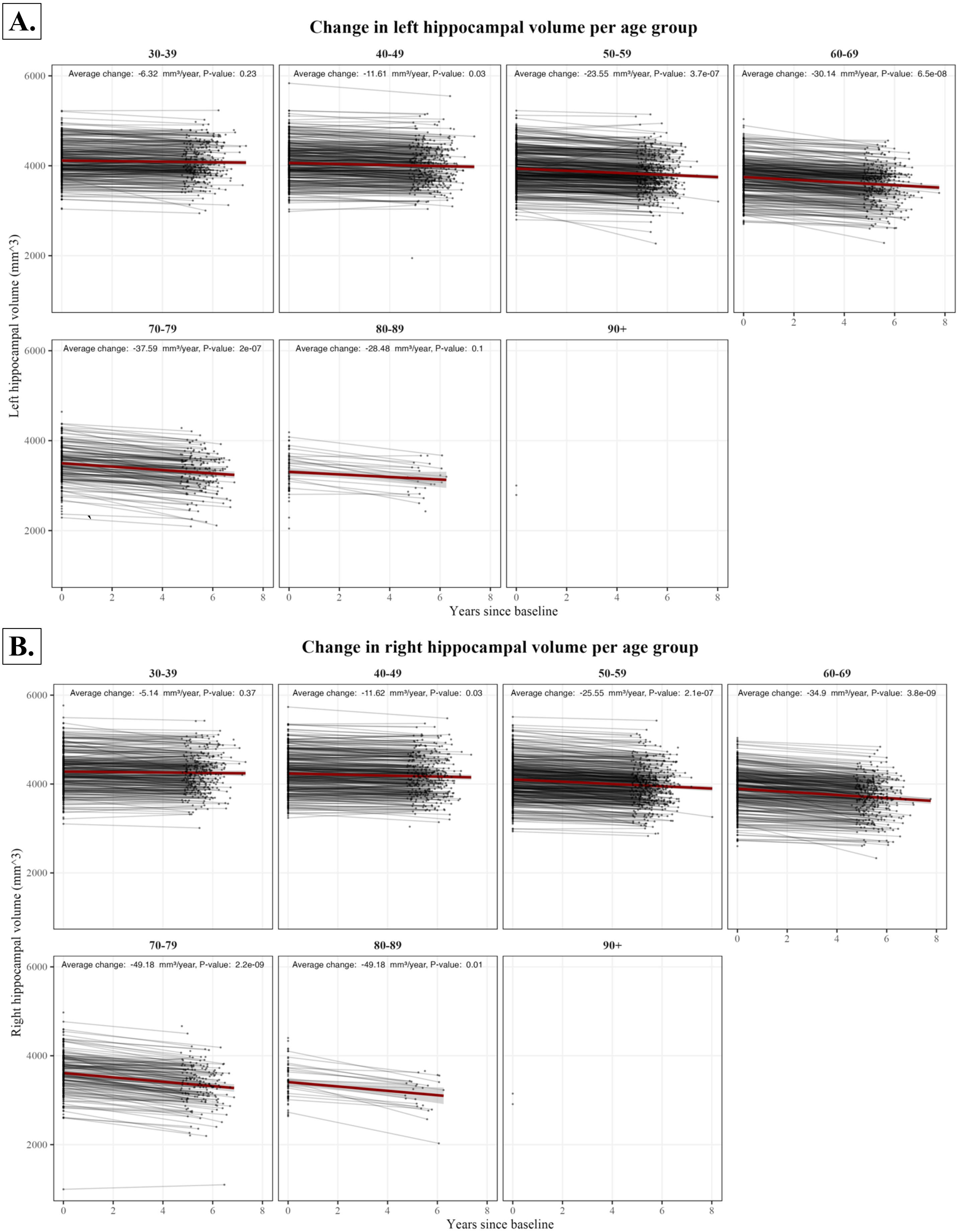

Supplementary Figure S3: Longitudinal change of brain imaging measures

C.

Change in hippocampal asymmetry per age group

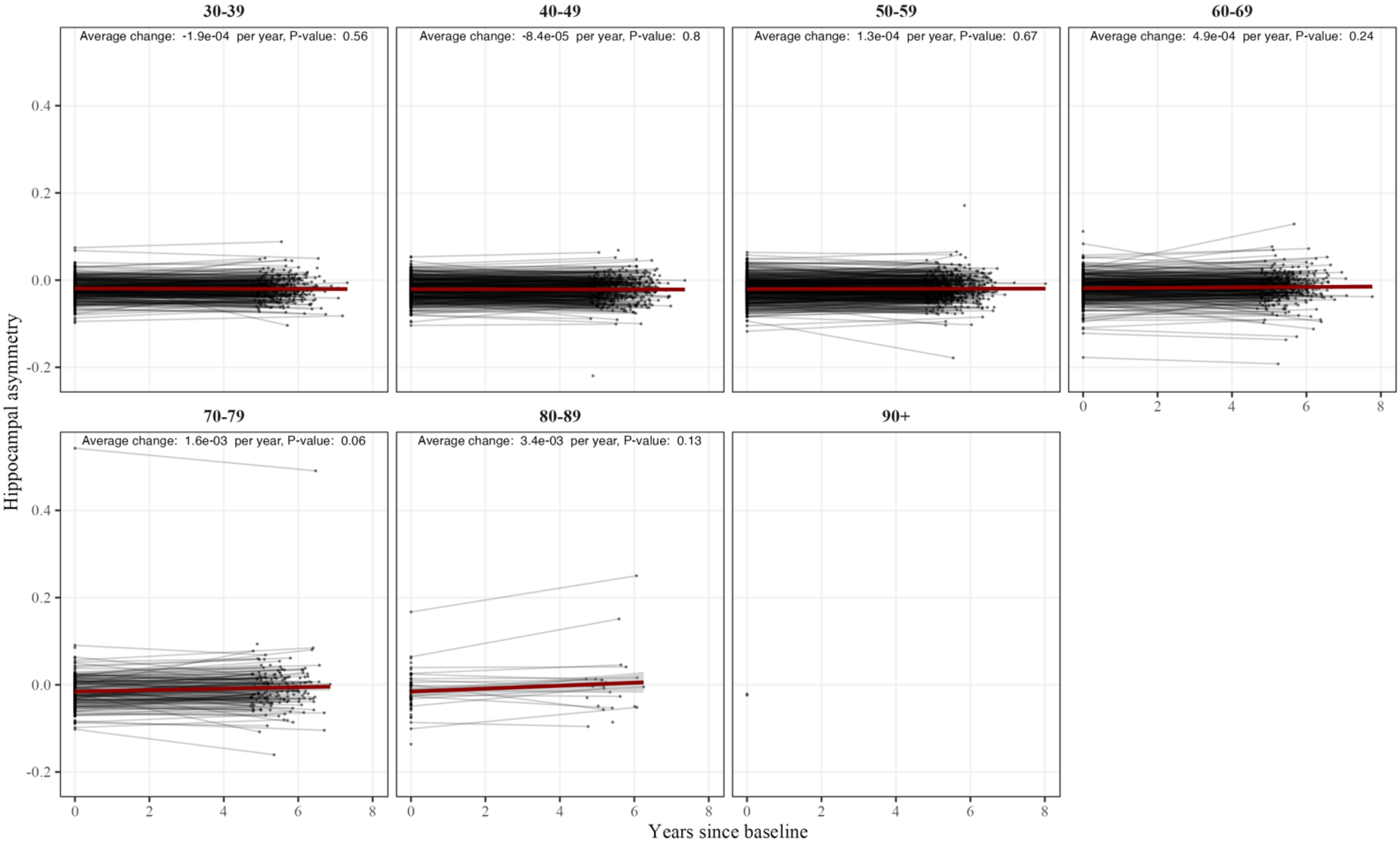

D.

Change in total brain volume per age group

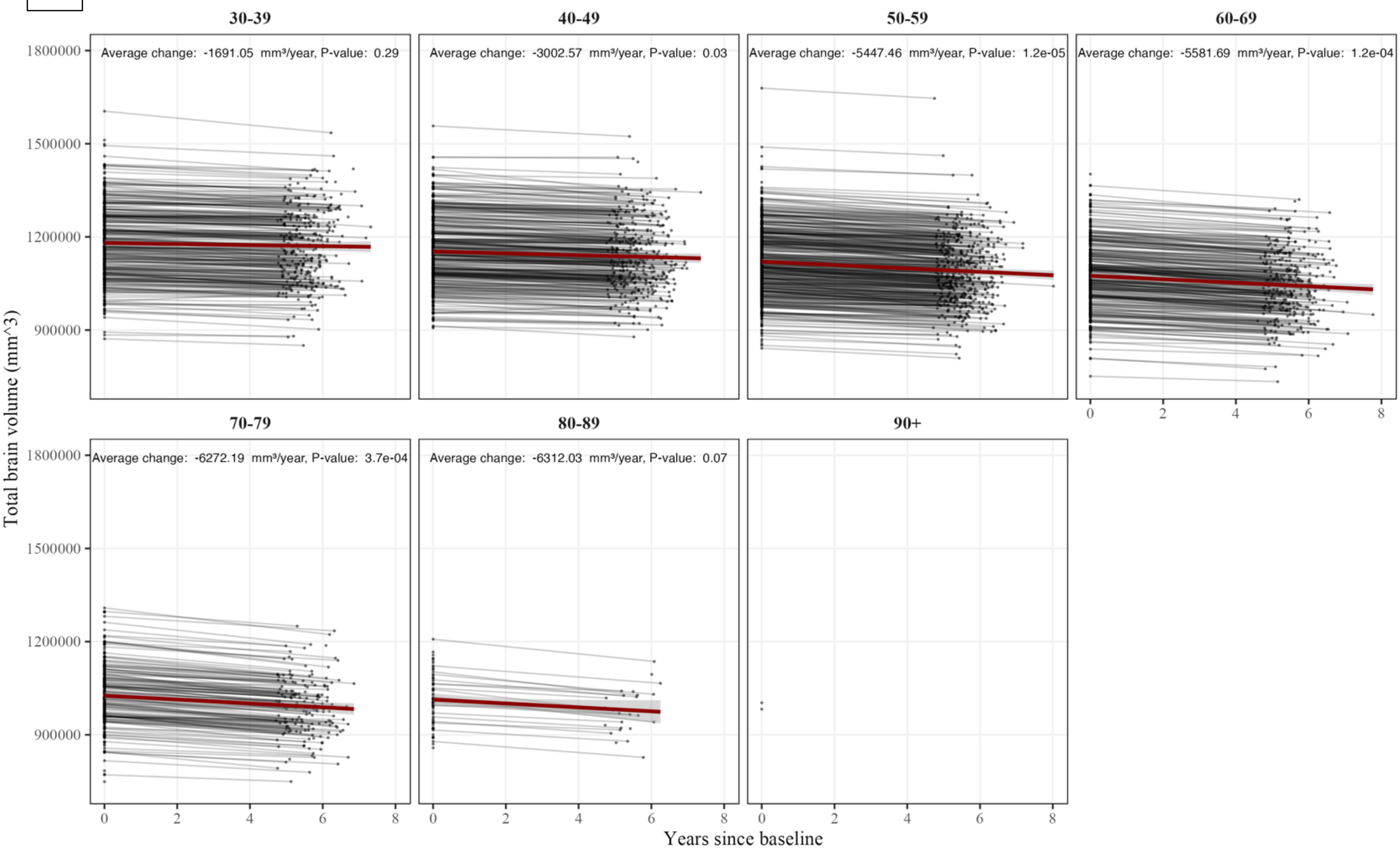

Supplementary Figure S3 (cont.): Longitudinal change of brain imaging measures
Each participant is represented by a single line connecting brain imaging measures as evaluated at baseline and the second round of examinations: left hippocampal volume (**A**), right hippocampal volume (**B**), hippocampal asymmetry (**C**), and total brain volume (**D**). Each dots represent one measurement. Participants have been stratified by age at baseline. The red line indicates the average change of participants in each stratum and the shaded area the corresponding 95% confidence interval. Average change rates and corresponding p-values have been calculated using linear regression

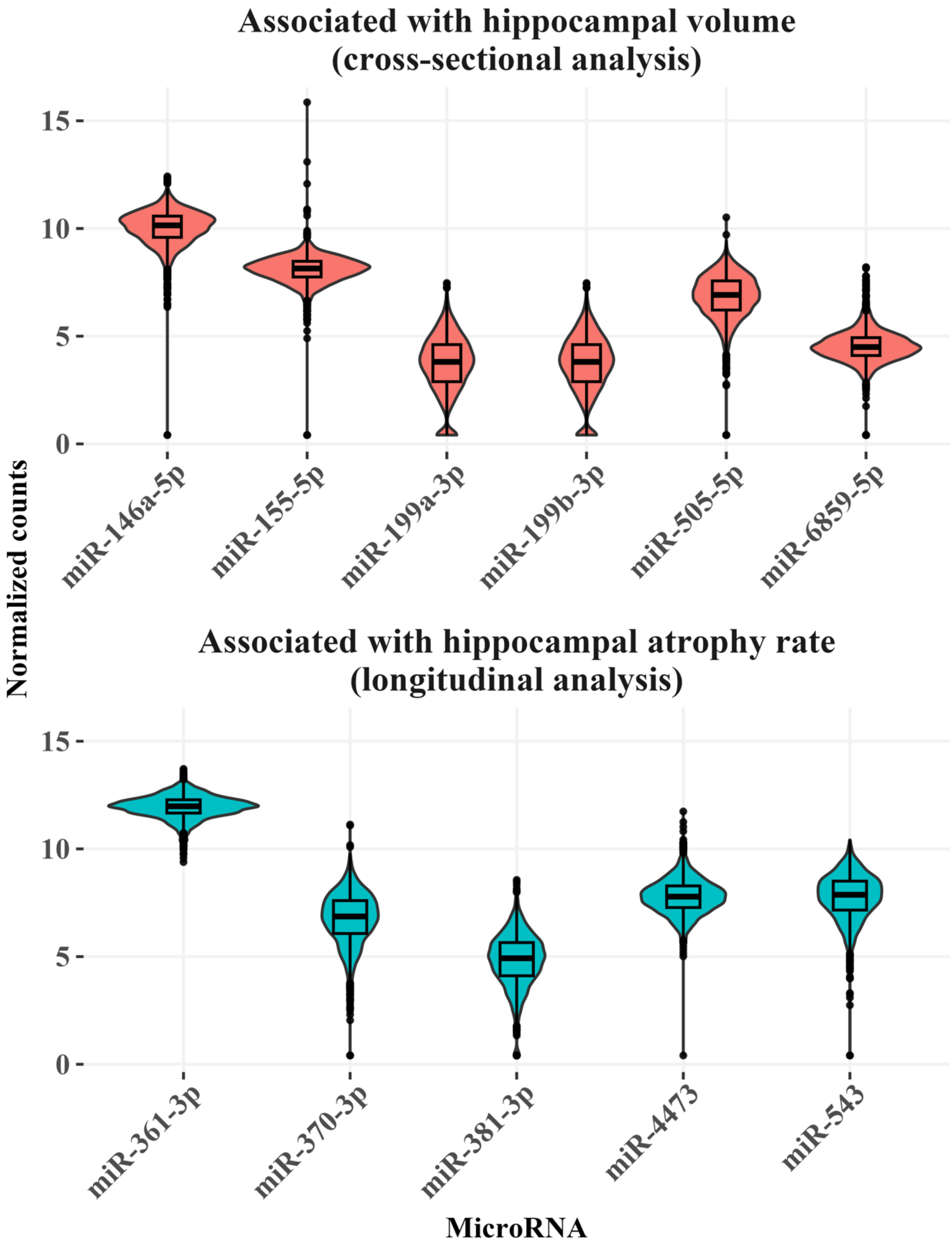

Supplementary Figure S4: Distribution of normalized counts of miRNAs associated with hippocampal volume and atrophy rate

The violin boxplots show the normalized and log-transformed counts for miRNAs identified in the cross-sectional or longitudinal analysis. The plots were created using all available miRNA data, regardless if brain imaging data was available.

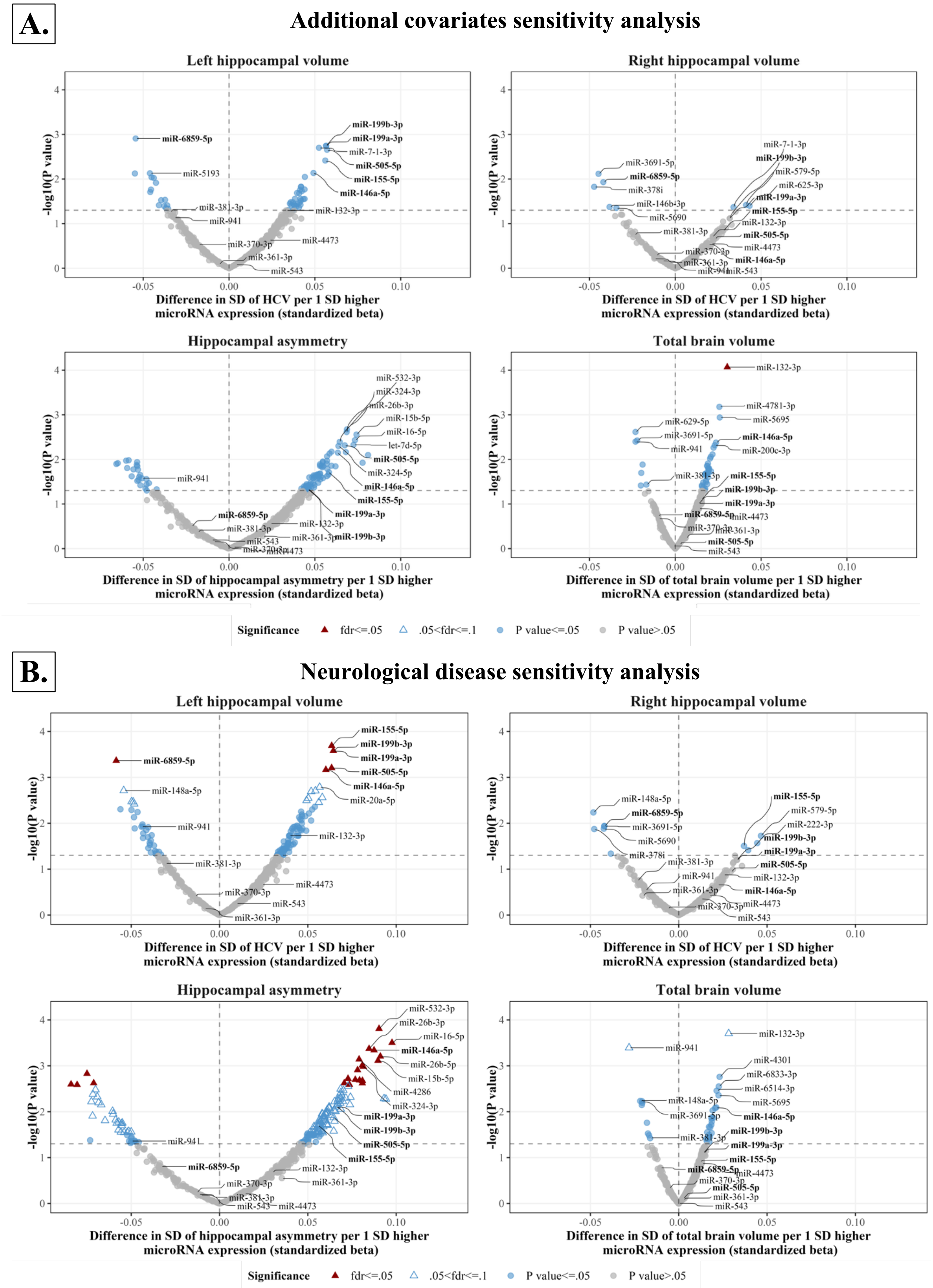

Supplementary Figure S5: Sensitivity analyses of the cross-sectional association of miRNAs with brain imaging measures.

The volcano plots show the association of miRNAs with brain imaging measures when adjusting for additional covariates (baseline blood cell counts, lipoproteins, and estimated Glomerular Filtration Rate) in addition to age, sex and technical variables (**A.**) and after removing participants diagnosed with dementia, Parkinson's disease, multiple sclerosis and hippocampal sclerosis (**B.**). MicroRNAs annotated with bold letters were significantly associated with HCV in the main analysis. The dashed horizontal lines indicate the uncorrected P value < 0.05 cut-off.

Abbreviations: SD, Standard Deviation; fdr, false discovery rate; HCV, Hippocampal Volume

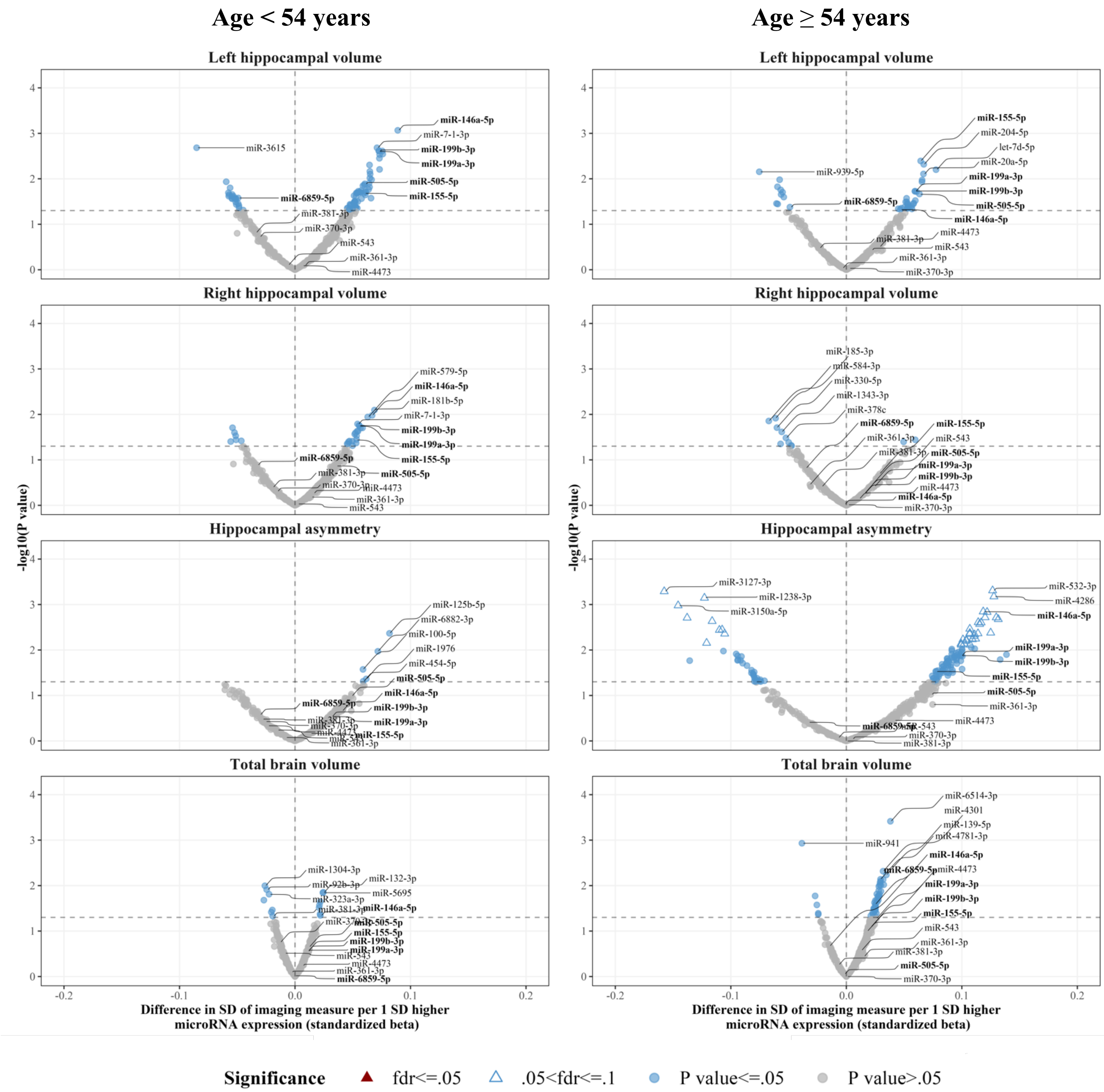

Supplementary Figure S6: Age stratification, cross-sectional analysis
The volcano plots show the association of miRNAs with brain imaging measures in younger (age < 54 years) and older (age ≥ 54 years) participants. MicroRNAs significantly associated with HCV in the main analysis which included all participants have been annotated with bold letters. The dashed horizontal lines indicate the uncorrected P value < 0.05 cut-off.
Abbreviations: HCV. Hippocampal Volume; SD, Standard Deviation; fdr, False Discovery Rate

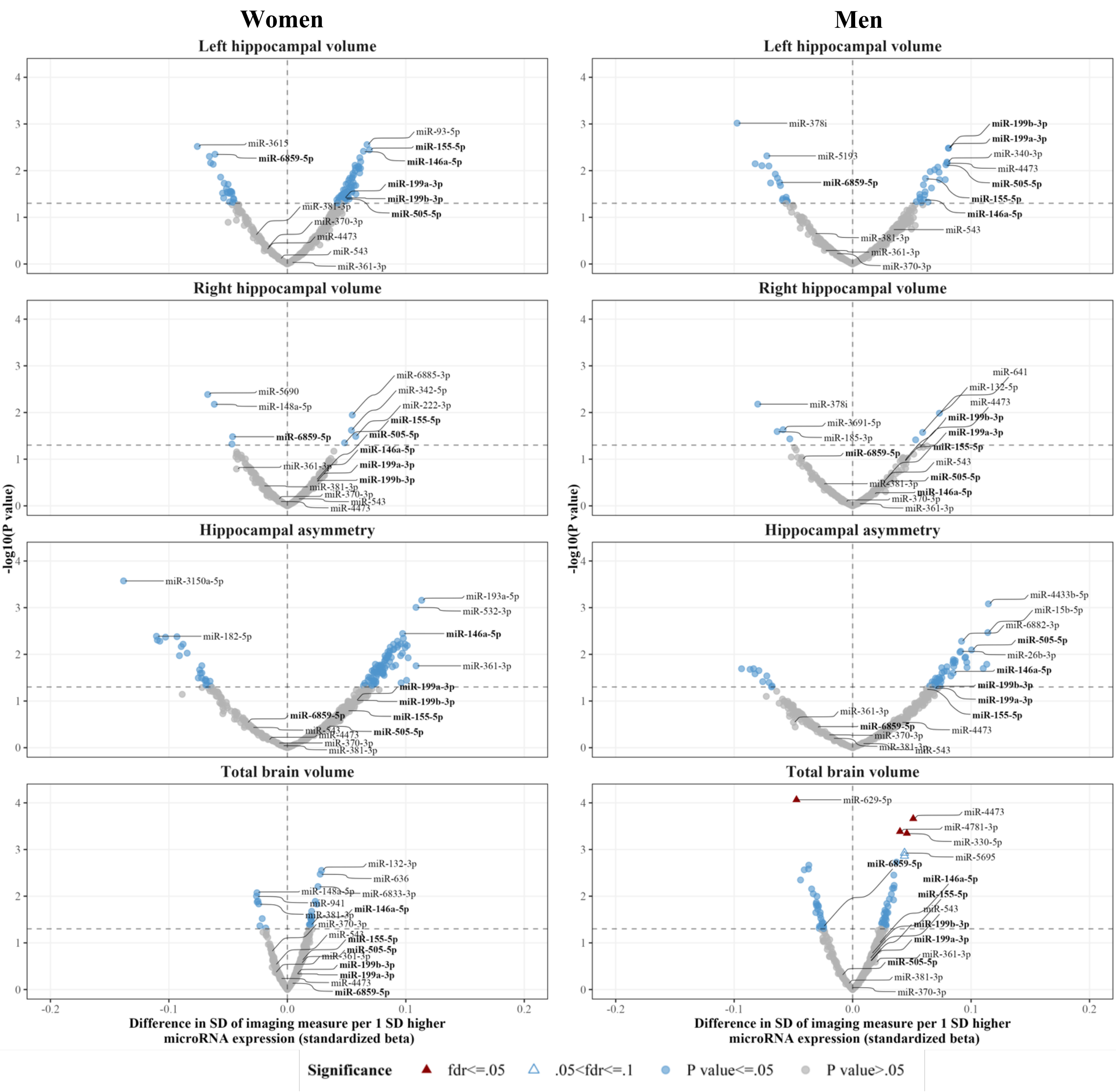

Supplementary Figure S7: Sex stratification, cross-sectional analysis
The volcano plots show the association of miRNAs with brain imaging measures in women and men. MicroRNAs significantly associated with HCV in the main analysis which included all participants have been annotated with bold letters. The dashed horizontal lines indicate the uncorrected P value < 0.05 cut-off.
Abbreviations: HCV. Hippocampal Volume; SD, Standard Deviation; fdr, False Discovery Rate

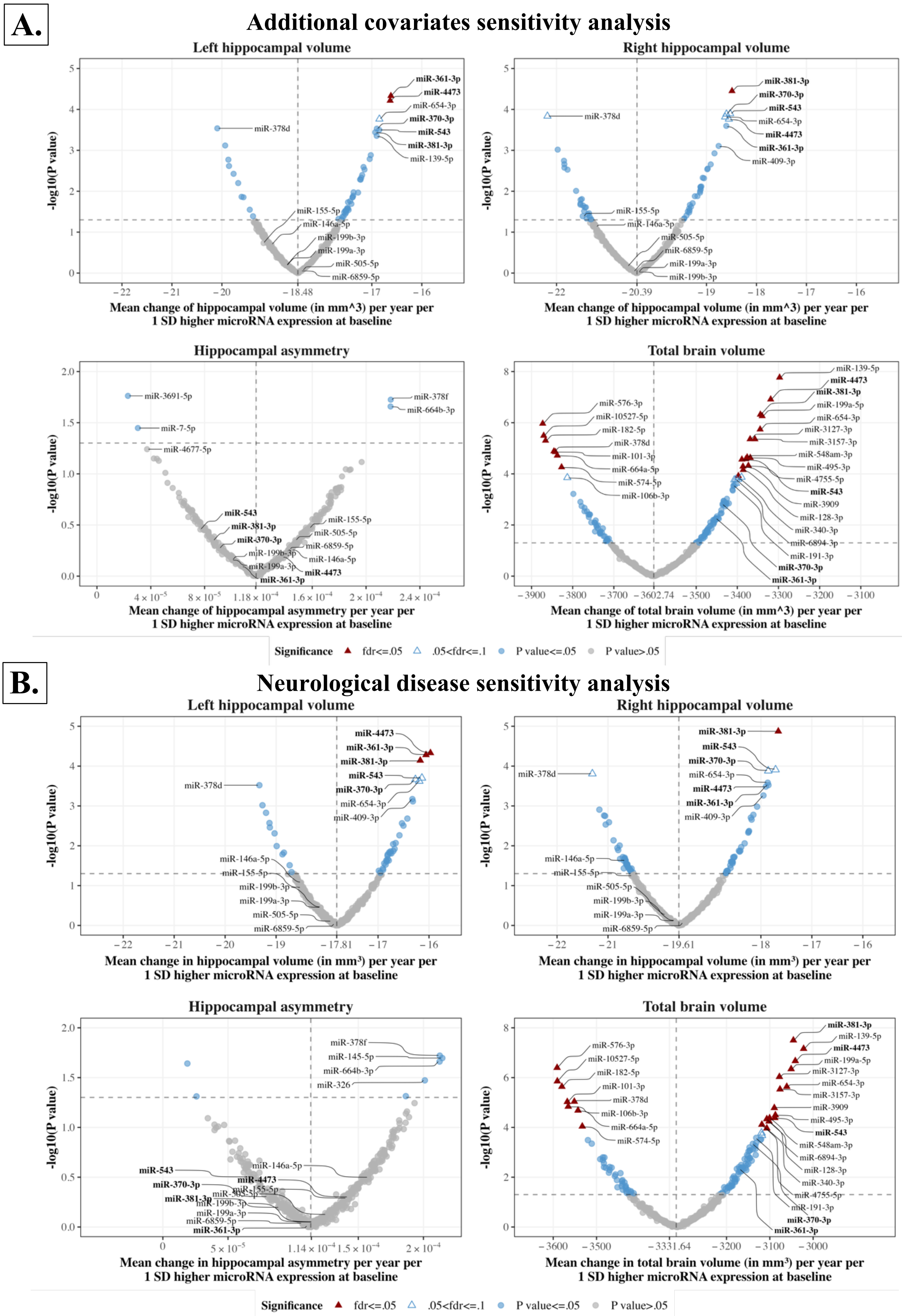

Supplementary Figure S8: Sensitivity analyses of the longitudinal association of miRNAs with brain imaging measures.

The volcano plots show the association of baseline miRNA expression with the rate of change of brain imaging measures when adjusting for additional covariates (baseline blood cell counts, lipoproteins, and estimated Glomerular Filtration Rate) in addition to age, sex and technical variables (**A.**) and after removing participants diagnosed with dementia, Parkinson's disease, multiple sclerosis and hippocampal sclerosis (**B.**). MicroRNAs annotated with bold letters were significantly associated with longitudinal hippocampal volume change rate in the main analysis. The dashed horizontal lines indicate the uncorrected P value < 0.05 cut-off. To facilitate plotting, different axis scales have been used for hippocampal volume change, asymmetry change, and total brain volume change.

Abbreviations: SD, Standard Deviation; fdr, false discovery rate

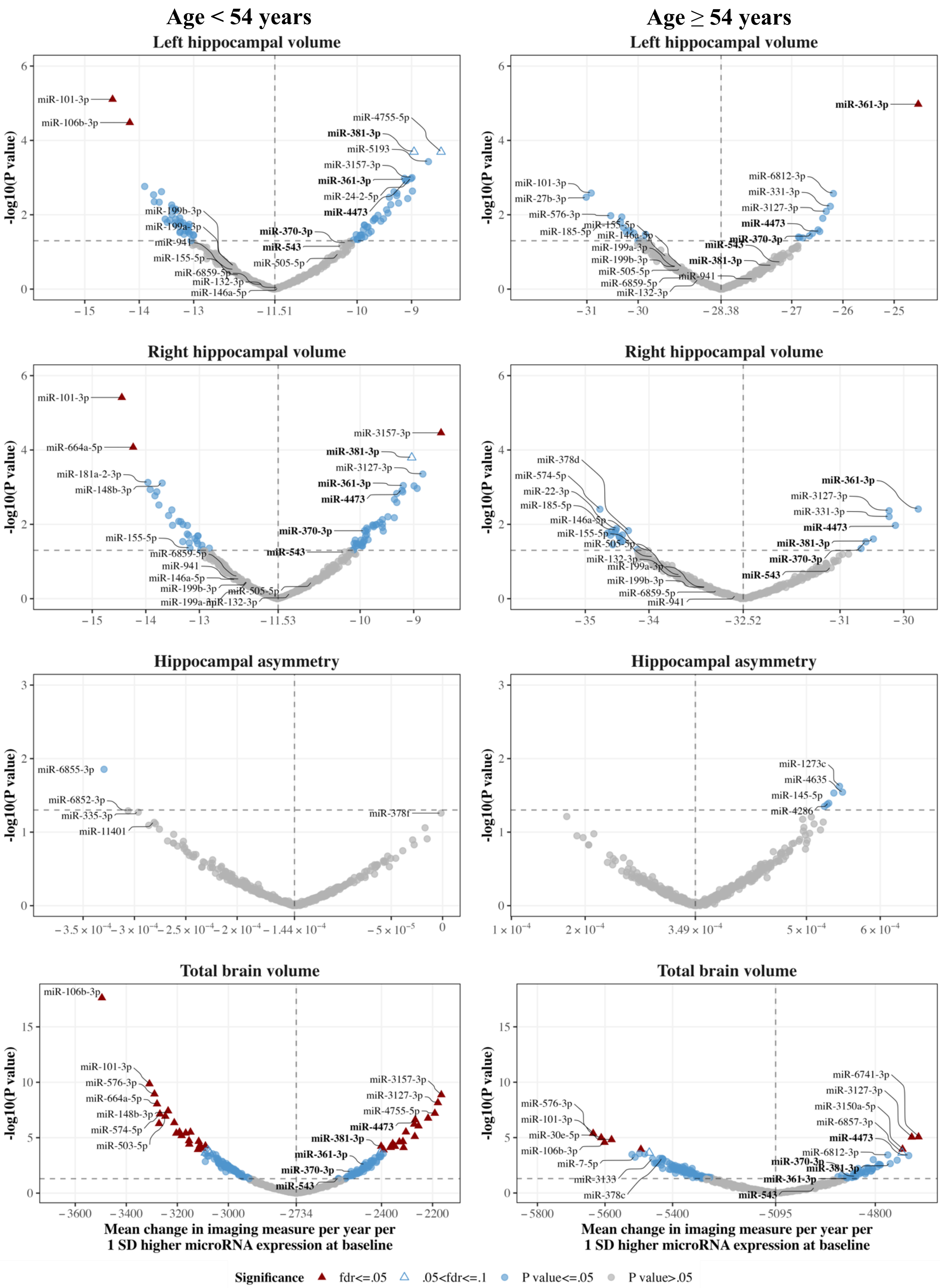

Supplementary Figure S9: Age stratification, longitudinal analysis

The volcano plots show the association of baseline miRNA expression with the yearly change of brain imaging measures in younger (age < 54 years at baseline) and older (age ≥ 54 years at baseline) participants. MicroRNAs significantly associated with hippocampal atrophy rate in the main analysis have been annotated with bold letters. The dashed horizontal lines indicate the uncorrected P value < 0.05 cut-off. Please note that, to facilitate plotting, different axis scales have been used.

Abbreviations: SD, Standard Deviation; fdr, False Discovery Rate

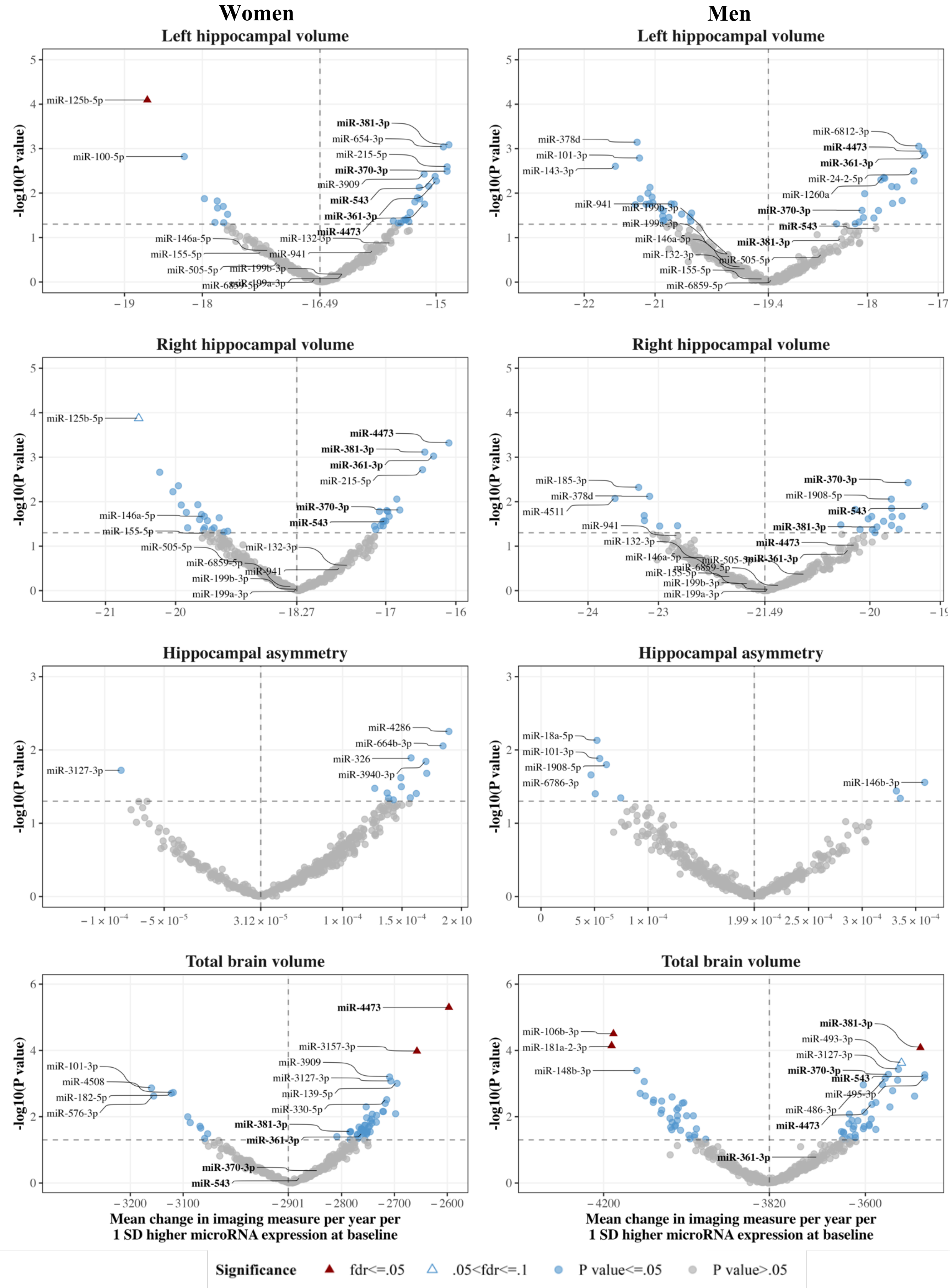

Supplementary Figure S10: Sex stratification, longitudinal analysis

The volcano plots show the association of baseline miRNA expression with the yearly change of brain imaging measures in women and men. MicroRNAs significantly associated with hippocampal atrophy rate in the main analysis have been annotated with bold letters. The dashed horizontal lines indicate the uncorrected P value < 0.05 cut-off. Please note that, to facilitate plotting, different axis scales have been used.

Abbreviations: SD, Standard Deviation; fdr, False Discovery Rate

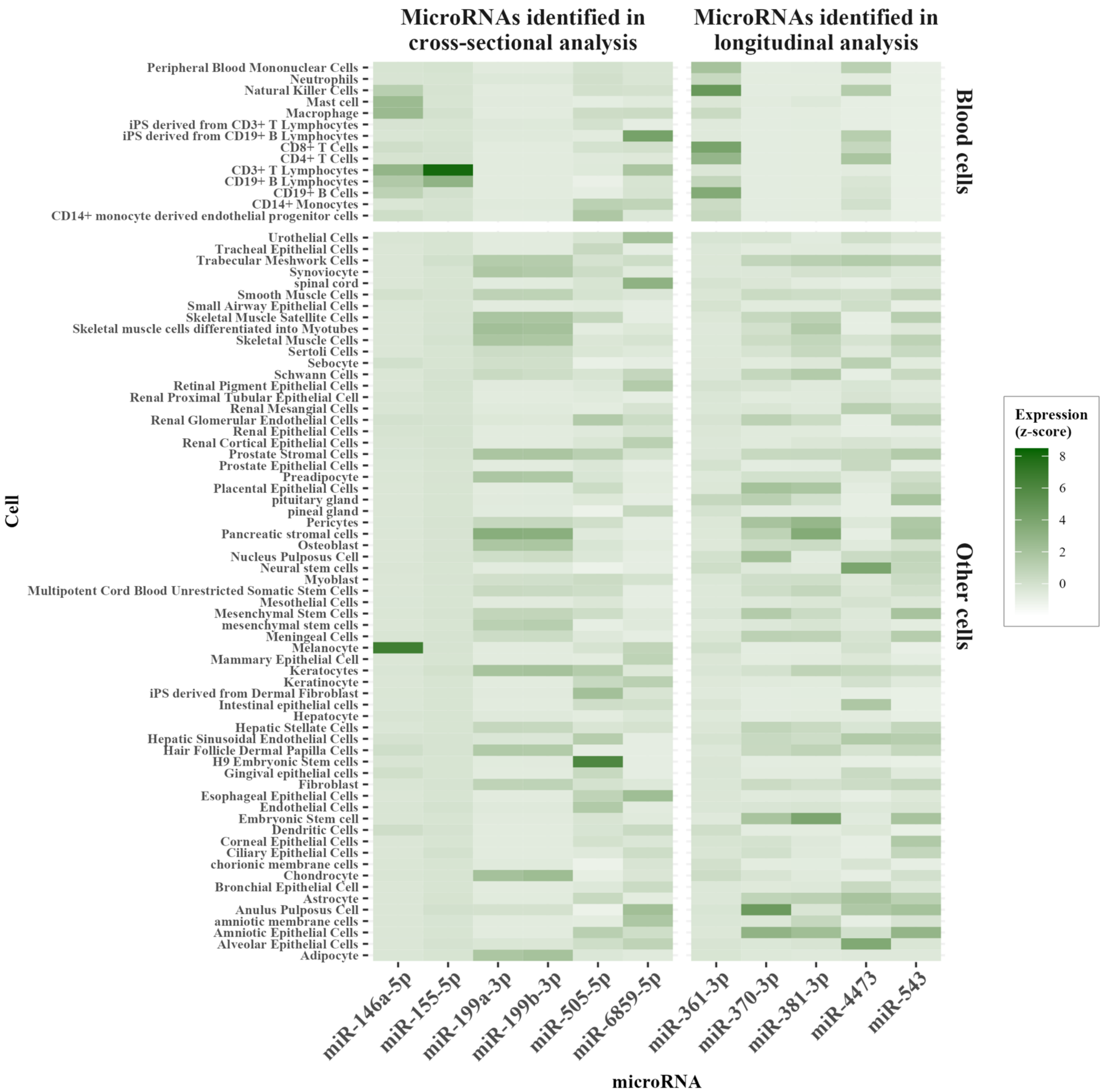

Supplementary Figure S11: MicroRNA expression in cells.
The heatmap shows relative miRNA expression (converted to a z-score for each miRNA) in various cell lines, for the miRNA associated with hippocampal volume cross-sectionally or longitudinally.

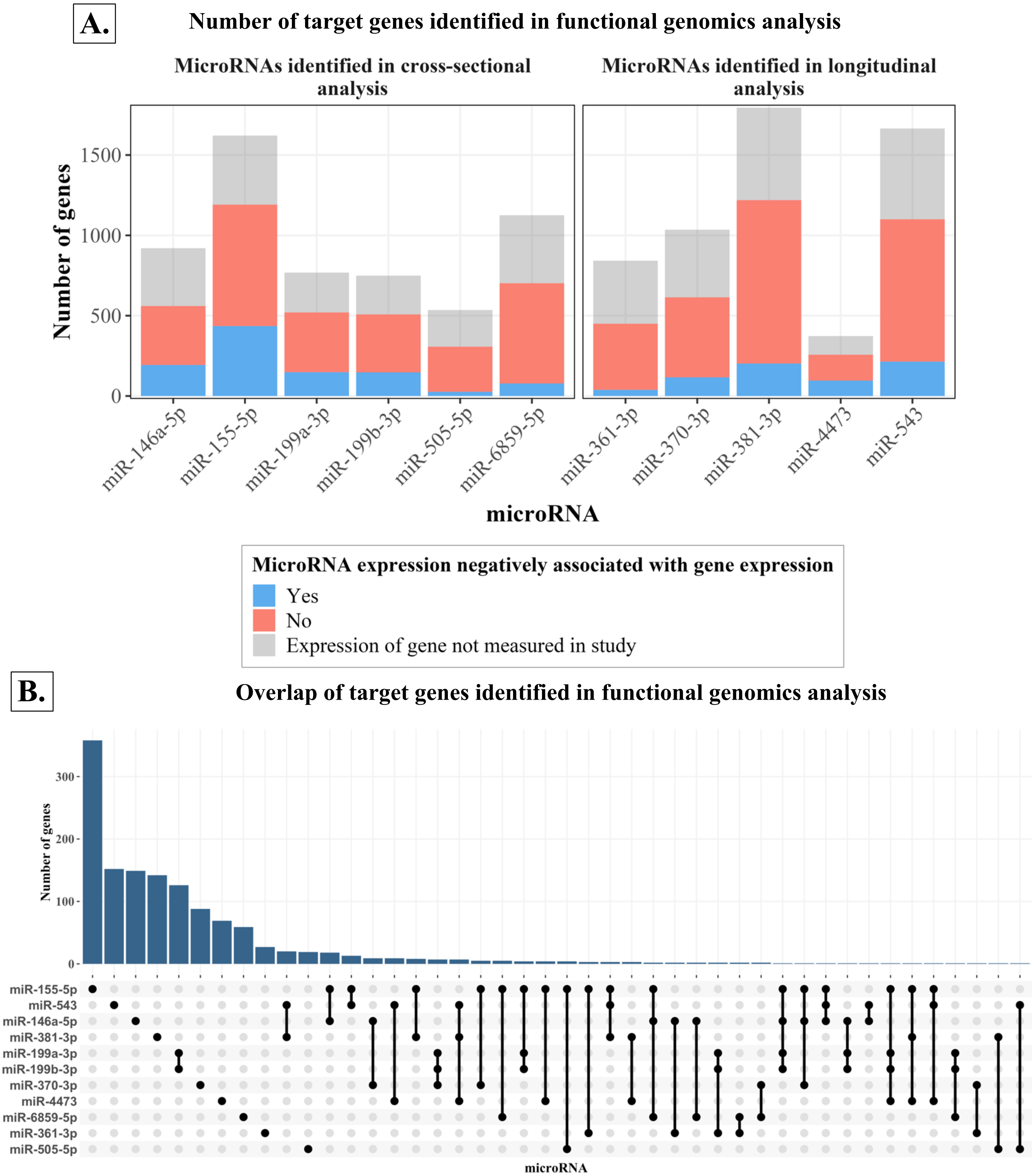

Supplementary Figure S12: MicroRNA target genes identified through functional genomics
The bar plot (**A**.) shows the number of genes that were computationally predicted to be targeted by hippocampus-related miRNAs, and the number of these predicted targets for which miRNA expression was negatively associated with gene expression. The association of miRNAs with some predicted target genes could not be examined, as expression of these genes was not measured in our study or was filtered out during quality control. These genes are shown with gray color. The upset plot (**B.**) shows the number and overlap of target genes that each miRNA was negatively associated with.

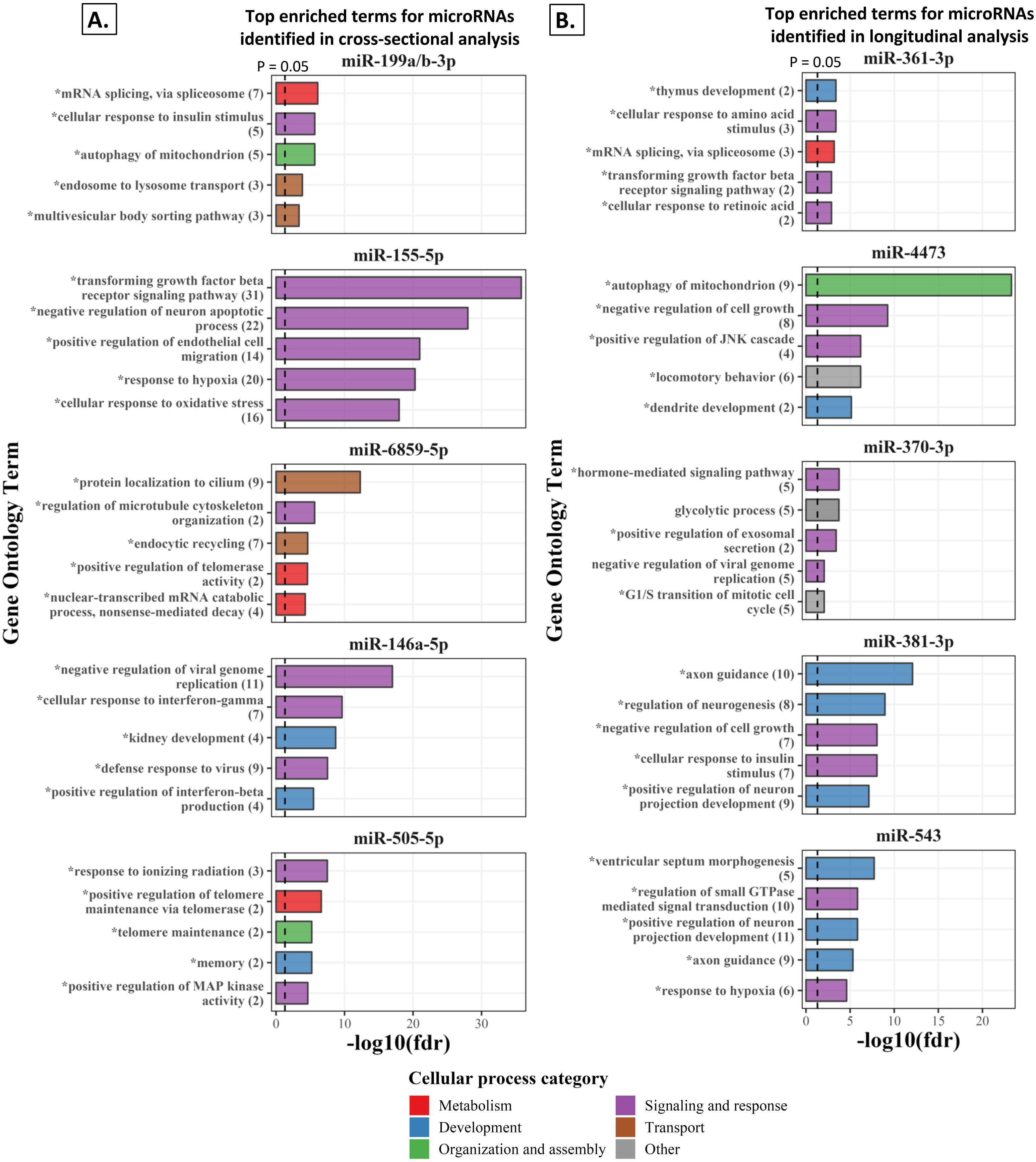

Supplementary Figure S13: Enrichment analysis of miRNA target genes expressed in the hippocampus. Results of *Gene ontology: Biological Process* enrichment analysis of miRNA target genes, filtering for genes with high expression in the hippocampus. The top 5 significantly enriched pathways (lowest p-value) are shown in the bar plots, for each miRNA associated with **A.** hippocampal volume cross-sectionally and **B.** hippocampal volume change rate longitudinally. Pathways marked with an asterisk **(*)** were enriched among target genes in the main analysis, without accounting for hippocampal expression. Significantly enriched pathways for each miRNA were grouped in broad categories, indicated by bar colors. Numbers in parentheses next to miRNA names indicate the total number of target genes of this miRNA that was included in *Gene Ontology*. P-values have been adjusted for multiple testing using the Benjamini-Hochberg false discovery rate (fdr) method.

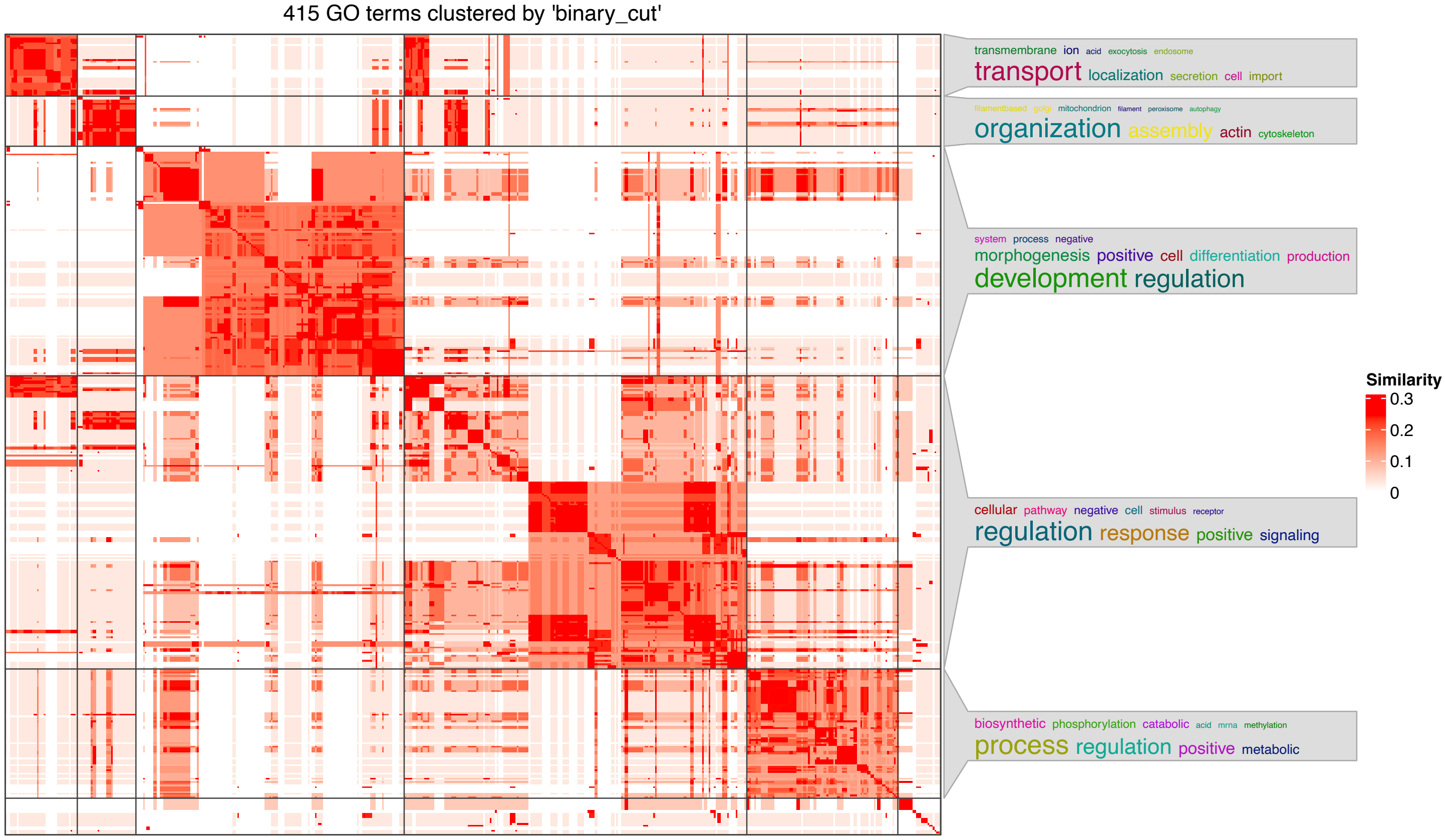

Supplementary Figure S14: Clustering of *Gene Ontology: Biological Processes* with the *SimplifyEnrichment* package. The heatmap shows the similarity matrix of terms, determined based on gene overlap. The word clouds show the most commonly appearing terms in each cluster, and were used to assign cluster names.

References

1. El Assaad MA, Topouchian JA, Asmar RG. Evaluation of two devices for self-measurement of blood pressure according to the international protocol: the Omron M5-I and the Omron 705IT. Blood Press Monit. 2003 Jun;8(3):127–33.

2. Coors A, Breteler MMB, Ettinger U. Processing speed, but not working memory or global cognition, is associated with pupil diameter during fixation. Psychophysiology. 2022 Nov;59(11):e14089.

3. Mueller ST, Piper BJ. The psychology experiment building language (PEBL) and PEBL test battery. J Neurosci Methods. 2014 Jan 30;222:250–9.

4. Boenniger MM, Staerk C, Coors A, Huijbers W, Ettinger U, Breteler MMB. Ten German versions of Rey's auditory verbal learning test: Age and sex effects in 4,000 adults of the Rhineland Study. J Clin Exp Neuropsychol. 2021 Aug;43(6):637–53.

5. Coors A, Merten N, Ward DD, Schmid M, Breteler MMB, Ettinger U. Strong age but weak sex effects in eye movement performance in the general adult population: Evidence from the Rhineland Study. Vision Res. 2021 Jan;178:124–33.

6. Ru Y, Kechris KJ, Tabakoff B, Hoffman P, Radcliffe RA, Bowler R, et al. The multiMiR R package and database: integration of microRNA-target interactions along with their disease and drug associations. Nucleic Acids Res. 2014 Jul 24;42(17):e133.

7. Huang H-Y, Lin Y-C-D, Li J, Huang K-Y, Shrestha S, Hong H-C, et al. miRTarBase 2020: updates to the experimentally validated microRNA-target interaction database. Nucleic Acids Res. 2020 Jan 8;48(D1):D148–54.

8. McGeary SE, Lin KS, Shi CY, Pham TM, Bisaria N, Kelley GM, et al. The biochemical basis of microRNA targeting efficacy. Science. 2019 Dec 20;366(6472).

9. Chen Y, Wang X. miRDB: an online database for prediction of functional microRNA targets. Nucleic Acids Res. 2020 Jan 8;48(D1):D127–31.

10. Liu W, Wang X. Prediction of functional microRNA targets by integrative modeling of microRNA binding and target expression data. Genome Biol. 2019 Jan 22;20(1):18.

11. Oliveira AC, Bovolenta LA, Nachtigall PG, Herkenhoff ME, Lemke N, Pinhal D. Combining Results from Distinct MicroRNA Target Prediction Tools Enhances the Performance of Analyses. Front Genet. 2017 May 16;8:59.

12. Wu T, Hu E, Xu S, Chen M, Guo P, Dai Z, et al. clusterProfiler 4.0: A universal enrichment tool for interpreting omics data. Innovation (Camb). 2021 Aug 28;2(3):100141.

13. Yu G, Wang L-G, Han Y, He Q-Y. clusterProfiler: an R package for comparing biological themes among gene clusters. OMICS. 2012 May;16(5):284–7.

14. Ashburner M, Ball CA, Blake JA, Botstein D, Butler H, Cherry JM, et al. Gene Ontology: Tool for the unification of biology. Nat Genet. 2000 May;25(1):25–9.

15. The Gene Ontology Consortium. The Gene Ontology resource: enriching a GOld mine. Nucleic Acids Res. 2021 Jan 8;49(D1):D325–34.

16. Sayols S. rrvgo: a Bioconductor package to reduce and visualize Gene Ontology terms. Bioconductor; 2020.

17. Gu Z, Hübschmann D. simplifyEnrichment: A Bioconductor Package for Clustering and Visualizing Functional Enrichment Results. Genomics Proteomics Bioinformatics. 2023 Feb;21(1):190–202.

18. Huan T, Rong J, Liu C, Zhang X, Tanriverdi K, Joehanes R, et al. Genome-wide identification of microRNA expression quantitative trait loci. Nat Commun. 2015 Mar 20;6:6601.

19. Watanabe K, Taskesen E, van Bochoven A, Posthuma D. Functional mapping and annotation of genetic associations with FUMA. Nat Commun. 2017 Nov 28;8(1):1826.

20. Hemani G, Zheng J, Elsworth B, Wade KH, Haberland V, Baird D, et al. The MR-Base platform supports systematic causal inference across the human phenome. eLife. 2018 May 30;7.

21. Smith SM, Douaud G, Chen W, Hanayik T, Alfaro-Almagro F, Sharp K, et al. An expanded set of genome-wide association studies of brain imaging phenotypes in UK Biobank. Nat Neurosci. 2021 May;24(5):737–45.

22. Brouwer RM, Klein M, Grasby KL, Schnack HG, Jahanshad N, Teeuw J, et al. Genetic variants associated with longitudinal changes in brain structure across the lifespan. Nat Neurosci. 2022 Apr 5;25(4):421–32.
